# *bla*_CTX-M-152_, a Novel Variant of CTX-M-group-25, Identified in a Study Performed on the Prevalence of Multidrug Resistance among Natural Inhabitants of River Yamuna, India

**DOI:** 10.3389/fmicb.2016.00176

**Published:** 2016-02-23

**Authors:** Mudsser Azam, Arif T. Jan, Qazi M. R. Haq

**Affiliations:** ^1^Microbiology Research Laboratory, Department of Biosciences, Jamia Millia IslamiaNew Delhi, India; ^2^Molecular Biology Laboratory, School of Biotechnology, Yeungnam UniversityGyeongsan, South Korea

**Keywords:** antibiotics, ESBL, mechanisms of resistance, polluted environment, resistance genes

## Abstract

Natural environment influenced by anthropogenic activities creates selective pressure for acquisition and spread of resistance genes. In this study, we determined the prevalence of Extended Spectrum β-Lactamases producing gram negative bacteria from the River Yamuna, India, and report the identification and characterization of a novel CTX-M gene variant *bla*_*CTX-M-152*_. Of the total 230 non-duplicate isolates obtained from collected water samples, 40 isolates were found positive for ESBL production through Inhibitor-Potentiation Disc Diffusion test. Based on their resistance profile, 3% were found exhibiting pandrug resistance (PDR), 47% extensively drug resistance (XDR), and remaining 50% showing multidrug resistant (MDR). Following screening and antimicrobial profiling, characterization of ESBLs (*bla*_TEM_ *and bla*_*CTX-M*_), and mercury tolerance determinants (*mer*P, *mer*T, *and mer*B) were performed. In addition to abundance of *bla*_*TEM-116*_ (57.5%) and *bla*_*CTX-M-15*_ (37.5%), bacteria were also found to harbor other variants of ESBLs like *bla*_*CTX-M-71*_ (5%), *bla*_*CTX-M-3*_ (7.5%), *bla*_*CTX-M-32*_ (2.5%), *bla*_*CTX-M-152*_ (7.5%), *bla*_*CTX-M-55*_ (2.5%), along with some non-ESBLs; *bla*_*TEM-1*_ (25%) and *bla*_OXY_ (5%). Additionally, co-occurrence of mercury tolerance genes were observed among 40% of isolates. *In silico* studies of the new variant, *bla*_*CTX-M-152*_were conducted through modeling for the generation of structure followed by docking to determine its catalytic profile. CTX-M-152 was found to be an out-member of CTX-M-group-25 due to Q26H, T154A, G89D, P99S, and D146G substitutions. Five residues Ser70, Asn132, Ser237, Gly238, and Arg273 were found responsible for positioning of cefotaxime into the active site through seven H-bonds with binding energy of -7.6 Kcal/mol. Despite small active site, co-operative interactions of Ser237 and Arg276 were found actively contributing to its high catalytic efficiency. To the best of our knowledge, this is the first report of *bla*_*CTX-M-152*_ of CTX-M-group-25 from Indian subcontinent. Taking a note of bacteria harboring such high proportion of multidrug and mercury resistance determinants, their presence in natural water resources employed for human consumption increases the chances of potential risk to human health. Hence, deeper insights into mechanisms pertaining to resistance development are required to frame out strategies to tackle the situation and prevent acquisition and dissemination of resistance determinants so as to combat the escalating burden of infectious diseases.

## Introduction

Extended spectrum β-lactamases (ESBLs), which have emerged in response to the widespread use of cephalosporins, represent the most diverse group of class A β-lactamases. With more than 220 variants of *bla*_TEM_ and 172 different *bla*_*CTX-M*_ variants reported to date, these active site serine β-lactamases (*bla*_TEM_ and *bla*_*CTX-M*_) represent the most prevalent β-Lactamases among members of Enterobacteriaceae (http://www.lahey.org/studies). By conferring higher hydrolytic activity, ESBLs exhibit a high level of resistance toward aminopenicillins (ampicillin or amoxicillin), carboxypenicillins (carbenicillin or tricarcillin), ureidopenicillins (piperacillin), cephalosporins (cephalothin, cephaloridine, and cefuroxime), oxi-imino cephalosporins (cefotaxime and ceftriaxone), cefepime, and cefpirome. Based on the broad host range and efficiency of conjugation, *bla*_TEM_ and *bla*_*CTX-M*_genes after several mobilization events via horizontal gene transfer, have now became part of plasmids among different bacterial species (Thomas and Nielsen, 2005; Barlow et al., 2008; Woodford et al., 2009).

The emergence of ESBL producing isolates have been often studied in clinics because they are commonly associated with outbreaks or sporadic infections (Kohlenberg et al., 2012; Walsh, 2013). However, a lot of studies have also been dedicated toward investigation of ESBL producers among microbial inhabitants of aquatic environments that receive a continuous influx of treated and untreated sewage (Prado et al., 2008; Chagas et al., 2011; Korzeniewska and Harnisz, 2013; Wellington et al., 2013). There are reports that suggest an additional selection effect by partially metabolized (10–90%) antibiotics used for human and animal health care in aquatic ecosystems (Kummerer, 2009; Harnisz, 2013). Concomitantly, the persistence of mercury in sewage has enabled bacteria to develop an array of resistance mechanisms based on clustering of genes either on the chromosome or plasmids (Jan et al., 2009; Jackson et al., 2011). Co-selection for metal and drug resistance determinants has resulted in conferring an advantage to bacteria that helps their survival in heavily polluted environments (Seiler and Berendonk, 2012; Zhou et al., 2015). Taken together, these findings highlight the necessity for controlling the emergence, and therefore, the dissemination of multi-drug and mercury resistance in bacteria through aquatic environments.

Antibiotic resistance has worsened in developing countries owing to compromised sanitary conditions, which exacerbate movement of genes via mobile genetic elements (Schlüter et al., 2007; Kelly et al., 2009; Knapp et al., 2010). The disease burden of India is highest among different countries of the world (GARP-India, 2011). In this regard, Study of the Monitoring of Antimicrobial Resistance Trends (SMART) revealed *E. coli* in being the most prevalent pathogen among the top five resistant gram-negative bacteria, causing 47.8% of intra-abdominal and 44.3% of urinary tract infections worldwide (Morrissey et al., 2013). In another resistance surveillance program carried out in India, Mendes et al. (2013) found that nearly 78% of *E. coli* and 64% of *Klebsiella spp.* carry ESBL determinants imparting resistance (Mendes et al., 2013). Despite these studies, there still lies paucity in the available information regarding prevalence of resistance determinants among bacteria and the pattern of antimicrobial resistance they exhibit. Hence, it becomes necessary to have an understanding of potential genetic variables that lead to acquisition of resistance and information regarding ESBL producers (ESBL^+^) among the microbial inhabitants of aquatic environments. The present study was conducted to investigate the prevalence of ESBL genes (*bla*_TEM_ and *bla*_*CTX-M*_) and the pattern of antimicrobial resistance among the natural inhabitants of river Yamuna, India, in order to have an insight of the resistance mechanisms that operate against β-lactam antibiotics. A novel variant of *bla*_*CTX-M*_ gene identified in the study was characterized to unravel its catalytic profile through *in silico* studies. As the river receives higher amount of discharges (both treated and untreated), it raises serious concerns as water from the River Yamuna after passing through different stages of treatment processes, is used for several domestic, industrial and agricultural purposes. In studies of Sehgal et al. (2012) and Malik et al. (2014), they report presence of higher amount of mercury along with other metals in the samples collected from river Yamuna, India (Sehgal et al., 2012; Malik et al., 2014). Taking this into account, an investigation of *mer* operon genes as a representation of tolerance to metals was carried out to determine its role in the selection and survival of these isolates in polluted environments. The present study provides useful information regarding occurrence of multidrug resistance among bacterial inhabitants of aquatic environment that exhibit broader risk for community infections. From the study, it becomes evident that polluted water bodies acts as a pool for the emergence of new variants; thereby highlights the need to carry out in depth studies preferably toward understanding the factors that led to transfer and as such acquisition of different determinants among bacteria in their natural habitats.

## Methodology

### Sample collection and screening for identification of bacteria

Water samples were collected from the 22 km Delhi stretch of the Yamuna River, starting from upstream of the Wazirabad barrage to downstream of the Okhla barrage. The Yamuna River primarily receives sewage (treated, partially treated, or untreated) from domestic and industrial settings. For this study, samples were collected aseptically during March 2012–August 2014 from 13 different sites ~100–150 m downstream of major drains discharging into the river. Soon after collection, samples were screened for the presence of bacterial isolates using nutrient agar and nutrient broth. Lactose fermenting Gram negative bacterial colonies were initially assessed based on their characteristic growth on MacConkey agar and Eosin Methylin Blue (EMB) agar followed by the IMViC standard biochemical tests (Clinical and Laboratory Standards Institute, 2010). Isolates from single site that were found to have same phenotypes such as growth characteristics, colony morphology, and resistance phenotypes, were excluded from the study. Further confirmation of all non-duplicate phenotypically identified bacterial isolates was carried out through analysis of the 16S rRNA gene. Subsequent to amplification of the desired (~685 bp) fragment that exhibit maximum variability across different groups of bacteria, sequencing reaction was carried out to get isolates correctly annotated with their respective group members. In case, similarity with more than one group was observed, they were re-sequenced for larger (~1250 bp) fragment size of the 16S rRNA gene for identification.

### Antibiotic susceptibility tests

After identification, screening for ESBL production was performed against third generation cephalosporins (ceftazidime, cefotaxime, and ceftriaxone) by the Kirby Bauer disc diffusion method using Mueller Hinton Agar (MHA) plates. Isolates with a zone diameter of ≤ 22, ≤ 27, and ≤ 25 mm corresponding to ceftazidime, cefotaxime, and ceftriaxone, respectively, were considered ESBL producers. An Inhibitor-Potentiation Disc Diffusion (IPDD) test was then performed by placing discs containing ceftazidime (30 μg) and cefotaxime (30 μg) alone and in combination with clavulanic acid (10 μg) 30 mm apart on MHA plates. A ≥5 mm increase in zone diameter around the disc with antibiotic plus clavulanic acid relative to the discs with antibiotics alone was considered positive for ESBL production (Clinical and Laboratory Standards Institute, 2012). *K. pneumonia* ATCC 700603 and *E. coli* ATCC 25922 were used as ESBL positive and negative controls, respectively.

The *in vitro* antimicrobial susceptibilities against 21 antibiotics belonging to 13 different classes (β-lactam, aminoglycosides, fluoroquinolones, polymixins, rifampicins, tetracyclines, and trimethoprim from HiMedia labs., India) were then investigated according to the Clinical Laboratory Standards Institute guidelines (Clinical and Laboratory Standards Institute, 2012). The minimum inhibitory concentrations (MICs) against ceftazidime (CAZ), ceftazidime + clavulanic acid (CAC), cefotaxime (CTX), and cefotaxime + clavulanic acid (CEC) were determined for all ESBL-producing isolates by the broth micro-dilution method using Luria Bertania broth. Results were interpreted according to the CLSI guidelines (Clinical and Laboratory Standards Institute, 2012).

### Determination of mercury tolerance among bacterial isolates

Screened isolates were checked for tolerance to mercury by streaking on luria agar plates containing 0.02 mg/L mercuric chloride (HgCl_2_). This value was ~10 times higher than its permissible limit of 0.002 mg/L for drinking water (US-EPA, http://water.epa.gov/drink/). Isolates showing tolerance to mercury were selected for MIC determination against various concentrations of mercuric chloride (0.02–20 mg/L) by the broth micro-dilution method using Luria Bertania broth. The MIC was defined as the lowest concentration of HgCl_2_ at which no growth was seen for the isolates in the culture media. *E. coli* ATCC 25922 was used as negative control.

### Detection of genes imparting resistance to bacteria

Screened bacterial isolates were analyzed for the presence of ESBLs (*bla*_TEM_ and *bla*_*CTX-M*_) and *mer* operon determinants (*mer*P, *mer*T, and *mer*B) conferring resistance to a broad range of antibiotics and mercury. Overnight grown cultures were used for isolation of genomic DNA by phenol-chloroform-isoamyl (PCI) method and plasmid DNA by alkaline lysis method using Qiagen kit. After retrieving information from NCBI, multiple sequence alignment using ClustalW option of the BioEdit program, was performed for selection of region to design gene specific primers. After procurement of gene specific primers (Table 1), amplification corresponding to full or partial length (*bla*_TEM_, *bla*_*CTX-M-1*_, *bla*_*CTX-M-25*_, *mer*B, *mer*T, and *mer*P) gene sequences were accomplished under the following cycle conditions: 94°C for 5 min (initial denaturation) followed by 30 cycles of denaturation at 94°C for 1 min, annealing at temperatures specific for each primer set (54.5–62°C) for 30 s and extension at 72°C for 1 min followed by final extension at 72°C for 10 min. Following purification using a QIA quick spin column (Qiagen Inc.), samples corresponding to different gene products were sequenced using an automated sequencer (ABI 1377) at Xcelris Lab (Gujarat, India). Additionally, isolates harboring the new identified variant (*bla*_*CTX-M-152*_) were analyzed for localization of the gene on chromosomal DNA and/or plasmid DNA by PCR. A reaction of 16S rRNA gene was used as control in case plasmid DNA was used as template for PCR to minimize chances of contamination of chromosomal DNA.

**Table 1:** **Sequences of primers used for the detection of different genes among ESBL^+^ isolates**.

**Gene type**	**Primer**	**Sequence**	**Product size (bp)**	**Reference**
16S rDNA	ID-F	5′-GGCGGACGGGTGAGTAATG- 3′	685	Designed primers for this study.
	ID-R	5′-ATCCTGTTTGCTCCCCACG- 3′		
	RRF	5′-GGCGGACGGGTGAGTAATG-3′	1250	
	RRR	5′-GAAGTCGGAATCGCTAGTAATCG-3′		
TEM	TEM-F	5′-ATGAGTATTCAACATTTCCGTGT- 3′	861	
	TEM-R	5′-TTA CCA ATG CTT AAT CAG TGA GG- 3′		
CTX-M	CTX-MF	5′-SCVATGTGCAGYACCAGTAA- 3′	480	
	CTX-MR	5′-GCTGCCGGTYTTATCVCC- 3′		
CTX-M-1	CM1F	5′-ATGGTTAAAAAATCACTGCGYCAGTTCACGC- 3′	875	
	CM1R	5′-TTACAAACCGTYGGTGACGATTTTAGCCG- 3′		
CTX-M-2	CM2F	5′-ATGATGACTCAGAGCATTCGCC- 3′	742	
	CM2R	5′-TCGTTGGTGGTGCCATAATCTCC- 3′		
CTX-M-8	CM8F	5′-AACGCACAGACGCTCTACC- 3′	517	
	CM8R	5′-GGGTAGCCCAGCCTGAAT- 3′		
CTX-M-9	CM9F	5′-ATGGTGACAAAGAGAGTGCAACGG- 3′	875	
	CM9R	5′-TTACAGCCCTTCGGCGATGATTC- 3′		
CTX-M-25	CM25F	5′-ATGATGAGAAAAAGCGTAAGGCGGG- 3′	876	
	CM25R	5′-TTAATAACCGTCGGTGACAATTCTGGC- 3′		
*mer*P	merP-F	5′-ATGAAGAAACTGTTTGCCTCC- 3′	276	
	merP-R	5′-TCACTGCTTGACGCTGGACG- 3′		
*mer*T	merT-F	5′-TTAATAGAAAAATGGAACGAC- 3′	351	
	merT-R	5′-ATGTCTGAACCACAAAACGGG- 3′		
*mer*B	merB-F	5′-ATGAAGCTCGCCCCATATATTTTAG- 3′	667	
	merB-R	5′-TCACGGTGTCCTAGATGACATGG- 3′		

### *In silico* analysis of variants

Sequences of different genes were analyzed for genetic relatedness corresponding to their respective group members followed by identification of variations using ClustalW in the BioEdit 5.0.9 sequence analysis software and the MEGA6 software program. The complete coding sequence (CDS) of novel variant represented here as *bla*_*CTX-M-152*_ of *Kluyvera georgiana* was translated using the Expasy translation tool (www.expasy.org/translate). However, to obtain insight into the relationship between amino acid substitutions and affinity for the substrate (cefotaxime), we conducted modeling and docking studies of the variant *bla*_*CTX-M-152*_with respect to its ligand, cefotaxime.

### Analysis of deleterious substitution by SIFT

Sorting Intolerant from Tolerant (SIFT), which predicts the phenotypic effect of amino acid substitutions on a protein, works on the principle of correlation of protein evolution with protein function (Ng and Henikoff, 2001, 2003). Here, we submitted a query in the form of protein sequences to detect the nature of substitutions. SIFT analysis was conducted by allowing the algorithm to search for homologous sequences using the default settings (SWISS-PROT 45 and TrEMBL 28 databases, median conservation score 3.00, remove sequences >90% identical to query sequence). This generated alignments with homologous sequences and assigned scores to each residue between 0 and 1 for evolutionary conservation and intolerance and tolerance to substitution. SIFT scores < 0.05 are predicted by the algorithm to be intolerant (deleterious amino acid substitutions), whereas scores >0.05 are considered tolerant (Ng and Henikoff, 2003). A higher tolerance index of a particular amino acid substitution is associated with a lower probable impact.

### Homology modeling for structure prediction of CTX-M-152

To retrieve potential structural templates for homology modeling of *bla*_*CTX-M-152*_, *bla*_*CTX-M*_sequences were searched using the protein data bank (PDB). Rather than finding a close associate, we found that crystal structures for PDB ID 1YLJ (CTX-M-9) and 1IYQ (Toho-1) showed the highest homology (85 and 86%) with that of *bla*_*CTX-M-152*_. Using these as a template for modeling of our protein (KJ461948) by I-TASSER (an online server for modeling), the best model was selected based on the highest C-score and lowest Z-score. Simultaneously, protein modeling was conducted manually using Modeller version 9.12. Upon selection of the best model with the lowest DOPE score, a comparative study of both models (obtained from I-TASSER and Modeller 9.12) with our template was performed. The model with the lowest RMSD was then selected for further studies. Verification of the selected model for structural constrains was conducted using a Ramachandran plot generated with the Rampage server tool (http://mordred.bioc.cam.ac.uk/~rapper/rampage). Prior to docking studies, the verified structure was submitted to the Dogsite Server (http://dogsite.zbh.uni-hamburg.de/) for potential active site prediction. This tool predicted the existence of 10 different pockets, and the one with the highest P score was selected as the most reliable active site pocket and considered to have potential active site residues.

### Docking of cefotaxime with CTX-M-152

Following retrieval of the cefotaxime structure from Drug Bank (Accession no. DB00493), it was docked against the modeled structure of CTX-M-152 using Autodock 4.2. Before docking, the ligand was prepared by adding partial Gasteiger charges and defining free rotatable bonds. Simultaneously, the target structure (CTX-M-152) was prepared by removing solvent (water) molecules and adding non-polar hydrogen atoms. An affinity grid was generated using the Autogrid program of the Autodock package with a defined spacing of 1 Å and grid size of 50 × 50 × 50 Å. pI was calculated using the Compute pI/Mw tool provided by Expasy and crosschecked by ProtParam.

## Results

### Screening for ESBL producing bacterial isolates

**Figure 1:**
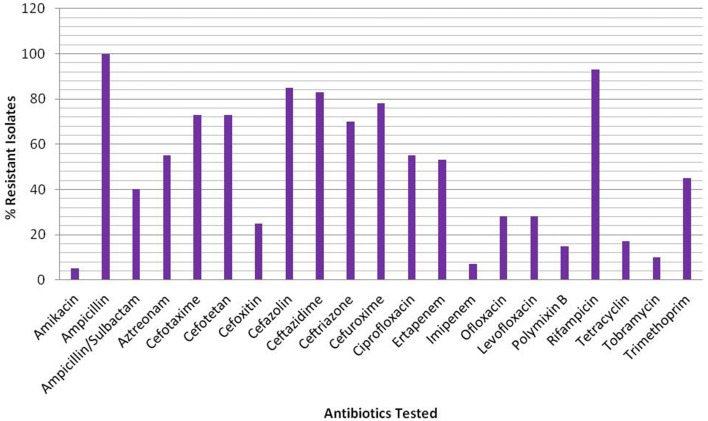
**Percentage of resistant isolates against the β-lactam and non β-lactam classes of antibiotics**.

Of the total 230 non-duplicate bacterial isolates, 40 isolates belonging to different groups of Gram negative bacteria were found to be ESBL producers based on the Kirby Bauer disc diffusion (Table S1) and IPDD tests (Table S2). Subsequent analysis of sequences corresponding to 16S rRNA genes from all 40 isolates for their correct representation revealed 34 to be *E. coli*, while the remaining isolates were *Klebsiella pneumonia* (1), *Aeromona sps*. (2), *Klebsiella oxytoca* (1), *K. georgiana* (1), and *Acinetobacter junii* (1) (Table 2). Screening of ESBL^+^ isolates revealed MIC values ≥512 mg/L for 25 (63%) isolates against cefotaxime, 17 (43%) isolates against ceftazidime, and 28 (70%) isolates against ceftriaxone (Table 2). Among the ESBL^+^ isolates, five (MRA11, MRC17, MRE18, MRE31, and MRE44) were resistant to a combination of ceftazidime + clavulanic acid and cefotaxime + clavulanic acid, and showed the highest resistance toward third generation cephalosporins. Resistance to fluoroquinolones was found to be increased with 55% (22/40) of isolates being resistant to ciprofloxacin and 28% (11/40) for levofloxacin and ofloxacin among ESBL^+^ isolates. The test isolates were reported to have high resistance level for ampicillin (100%) and rifampicin (93%), while on other hand, the resistance level was low for amikacin (5%) and imipenem (8%) (Figure 1).

**Table 2:** **Characterization of gene sequences along with their MIC values for different antibiotics and heavy metals among ESBL^+^ bacterial isolates**.

	**β-Lactamases genes**		**GenBank Accession nos**.		***mer*** **operon genes**			**MIC values (**μ**g/ml)**					
	**ESBL**	**Non-ESBL**	**16S rRNA gene**	**β-lactamases genes**	***mer*P**	***mer*T**	***mer*B**	**CAZ**	**CAC**	**CTX**	**CEC**	**CTR**	**HgCl_2_**
*Klebsiella pneumoniae* MRA3	TEM-116	–	KJ906614		−	+	−	>512	0.38	512	0.125	>512	2
*Aeromonas sps* MRA5	TEM-116, CTX-M-15	–	KJ957158	KJ923000, KM873149	+	+	+	>512	0.75	512	0.25	128	20
*Aeromonas sps* MRA10	TEM-116, CTX-M-15	–	KJ957159	KJ923001, KM873161	−	−	−	4	1	16	0.75	4	2
*E. coli* MRA11	TEM-116	TEM-1	KJ957160	KJ923003, KJ923002	+	+	+	512	>4	512	>1	>512	2
*Klebsiella oxytoca* MRA13	TEM-116	–	KJ957161	KJ923004	+	+	+	64	1.5	32	0.25	4	20
*E. coli* MRB2	TEM-116	–	KJ906615	KJ923005	+	+	−	256	2	64	0.5	64	2
*E. coli* MRB6	TEM-116, CTX-M-71	–	KJ906619	KJ923006, KM873170	+	+	+	32	0.5	>512	0.75	>512	20
*E. coli* MRC2	TEM-116, CTX-M-15	–	KJ906616	KJ923008, KM873150	+	+	−	16	2	128	0.5	512	20
*E. coli* MRC3	TEM-116, CTX-M-15	–	KJ906617	KJ923009, KM873151	−	−	−	32	1	512	0.5	>512	20
*E. coli* MRC6	TEM-116, CTX-M-71	–	KJ906618	KJ939551, KM873171	+	+	+	16	3	512	0.75	>512	2
*E. coli* MRC7	–	–	KM822763		+	−	−	32	0.25	16	0.125	16	2
*E. coli* MRC13	TEM-116	–	KM822764	KM873145	−	−	−	4	2	512	0.094	>512	2
*E. coli* MRC17	CTX-M-55	TEM-1	KJ906623	KM873174, KJ939552	+	+	−	256	>4	128	>1	256	20
*E. coli* MRC24	–	–	KM822765		−	−	−	>512	0.125	>512	0.094	128	2
*E. coli* MRE2	TEM-116, CTX-M-15	–	KC963022	KJ939553, KM873162	+	+	−	512	3	>512	0.125	512	2
*E. coli* MRF6	–	TEM-1	KC963027	KJ939560	+	+	+	>512	0.25	>512	0.032	512	20
*Acinetobacter junii* MRH8	CTX-M-15	TEM-1	KC963028	KM873163, KM593699	−	−	+	>512	3	256	0.023	16	2
*E. coli* MRK28	TEM-116	OXY	KJ923019	KM593700	+	+	−	512	1	128	0.125	512	2
*E. coli* MROB6	CTX-M-3	TEM-1	KC963015	KM873169, KM593701	−	+	+	64	0.5	16	0.125	16	20
*E. coli* MROB11	CTX-M15	TEM-1	KC963018	KM873164, KM593702	+	+	+	512	0.5	128	0.125	16	20
*E. coli* MROB16	CTX-M-15	TEM-1	KC963017	KM873165, KM593703	−	−	+	256	0.38	128	0.19	64	20
*E. coli* MRAE2	TEM-116, CTX-M-32	–	KM822766	KM593704, KR560052	−	−	−	16	2	>512	0.25	>512	2
*E. coli* MRAE5	TEM-116, CTX-M-15	–	KJ906620	KJ939554, KM873152	−	−	+	8	0.5	512	0.032	>512	2
*E. coli* MRAE6	TEM-116, CTX-M-15	–	KJ906624	KJ939555, KM873153	−	+	−	16	3	>512	0.19	>512	2
*E. coli* MRAE9	CTX-M-15	–	KJ923010	KM873154	+	+	−	16	0.5	128	0.094	>512	20
*E. coli* MRAE14	TEM-116	–	KM822767	KM873146	+	−	−	16	0.75	512	0.125	>512	2
*E. coli* MRAE17	CTX-M-3	–	KJ906621	KM873166	+	+	+	16	2	512	0.094	>512	2
*E. coli* MRAE18	TEM-116, CTX-M-3	–	KJ906622	KJ939556, KM873167	+	+	+	>512	>4	>512	>1	>512	2
*E. coli* MRAE21	TEM-116, CTX-M-152	–	KJ923011	KM873147, KM873172	+	+	−	>512	2	>512	0.064	>512	2
*E. coli* MRAE23	CTX-M-15	TEM-1	KJ923012	KM873155, KM593705	−	+	+	>512	0.38	>512	0.125	>512	20
*E.coli* MRAE25	–	–	KJ923013		+	+	+	>512	1	>512	0.094	>512	2
*E. coli* MRAE26	TEM-116	–	KJ923014	KJ939557	+	+	+	>512	0.094	>512	0.125	>512	20
*E. coli* MRAE27	TEM-116, CTX-M-15	–	KJ923015	KJ939558, KM873156	−	+	−	512	0.38	256	0.125	512	2
*E. coli* MRAE31	CTX-M-15	–	KJ923016	KM873157	+	+	+	>512	>4	>512	>1	>512	20
*E. coli* MRAE32	TEM-116, CTX-M-3, CTX-M-15	–	KJ923017	KJ939559, KM873168, KM873158	+	+	+	8	0.5	512	0.125	>512	2
*E. coli* MRAE33	TEM-116	–	KM822768	KM873148	+	+	+	64	0.38	512	0.047	>512	20
*E. coli* MRAE36	CTX-M-15	–	KJ957162	KM873159	+	+	+	16	0.25	256	0.125	>512	20
*E. coli* MRAE42	CTX-M-15	–	KJ957163	KM873160	−	−	−	64	1	512	0.094	>512	2
*E. coli* MRAE44	TEM-116, CTX-M-152	–	KJ923018	KM593706, KM873173	+	+	−	512	>4	256	>1	512	2
*Kluyvera georgiana* MRB7	TEM-116, CTX-M-152	–	KM822769	KJ923007, KJ461948	+	−	−	256	1.5	>512	0.38	256	2
*Klebsiella pneumoniae* ATCC 700603	–	–	–	–	−	−	−	>32	2	8	0.75	16	0.02
*E. coli* ATCC 25922	–	–	–	–	−	−	−	2	0.094	1	0.023	0	0.02

CAZ, Ceftazidime; CAC, Ceftazidime + clavulanic acid; CTX, Cefotaxime; CEC, Cefotaxime + clavulanic acid; CTR, Ceftriaxone; HgCl_2_, Mercuric chloride.

Based on European Centre for Disease Prevention and Control (ECDC) and the Centre for Disease Control and Prevention (CDC) categorization, 3% of the 40 isolates investigated in this study were PDR, 47% XDR and 50% MDR. Regardless of the origin or mode of action, resistance was observed for all classes of antibiotics among all isolates, with susceptibility slightly higher for polymixin B and tobramycin. Information regarding patterns of resistance for these isolates against different classes of antibiotics is summarized in Tables S3A,B. All bacterial isolates in the test library were multidrug resistant against at least four classes of antibiotics, with one strain represented as PDR being resistant to at least one agent of all 13 categories.

### Mercury tolerance among ESBL^+^ isolates

Screening of ESBL^+^ isolates for their ability to tolerate various concentrations of mercury revealed that 40% of isolates were tolerant to 2 mg/L (10^3^ times higher than its permissible limit of 0.002 mg/L for drinking water), while the rest were tolerant to 0.2 mg/L of mercury. Around, 23% isolates tolerating high concentrations of mercuric chloride (2 mg/L), were found positive for presence of all three (*mer*T, *mer*P, and *mer*B) *mer* operon genes. Presence of *mer* operon genes are believed for attributing bacterial isolates with the resistance phenotype. To our surprise, an *E. coli* isolate (MRC3) that tested negative for all three *mer* operon genes, was also found to tolerate high concentration of mercury (2 mg/L), attributed to the presence of resistance determinants other than that used in the study.

### Screening for determinants imparting dual resistance to bacteria

β-lactamase genes detected more often among the ESBL^+^ isolates include *bla*_*TEM-116*_ (23, 57.5%) and *bla*_*CTX-M-15*_ (15, 37.5%). Additionally, they were found to harbor *bla*_*CTX-M-71*_ (2, 5%), *bla*_*CTX-M-3*_ (3, 7.5%), *bla*_*CTX-M-32*_ (1, 2.5%), *bla*_*CTX-M-152*_ (3, 7.5%), *bla*_*CTX-M-55*_ (1, 2.5%), and some non-ESBL genes like *bla*_*TEM-1*_ (10, 25%) and *bla*_OXY_ (2, 5%) (Table 2). All of the CTX-M encoding isolates harbored genes with sequence homology to members of group 1 (i.e., CTX-M-15, 3, 32, 55, and 71), except for one sequence [later denoted *bla*_*CTX-M-152*_ by lahey's organization (www.lahey.org)] that showed maximum similarity to a CTX-M-group-25 member, *bla*_*CTX-M-78*_ (Rodríguez et al., 2010). The *mer* operon consist of genes mostly associated with functions such as transport (*mer*T and *mer*P), regulation (*mer*R and *mer*D), and reduction (*mer*A and *mer*B; Jan et al., 2009). Of 40 ESBL^+^ isolates, 26 (65%) and 28 (70%) were positive for *mer*P and *mer*T genes respectively that encodes for membrane transport proteins. The *mer*B gene encoding organomercurial lyase, which catalyzes protonolytic cleavage of the carbon-mercury bond of organomercurials, was amplified from 20 (50%) isolates (Table 2). Taken together, 14 bacterial isolates were found harboring broad spectrum *mer* operon genes (*mer*T, *mer*P, and *mer*B), known for their significant contribution in achieving tolerant phenotype against mercury. One isolate was found to have only *mer*B gene. In comparison to isolate MRC24 which was found negative for all genes, five isolates were found to harbor both ESBL genes (*bla*_TEM_ and *bla*_*CTX-M*_) and determinants of the *mer* operon (*mer*P, *mer*T, and *mer*B) responsible for imparting resistance to a broad range of antibiotics and mercury.

### Comparative studies of CTX-M variants

**Figure 2:**
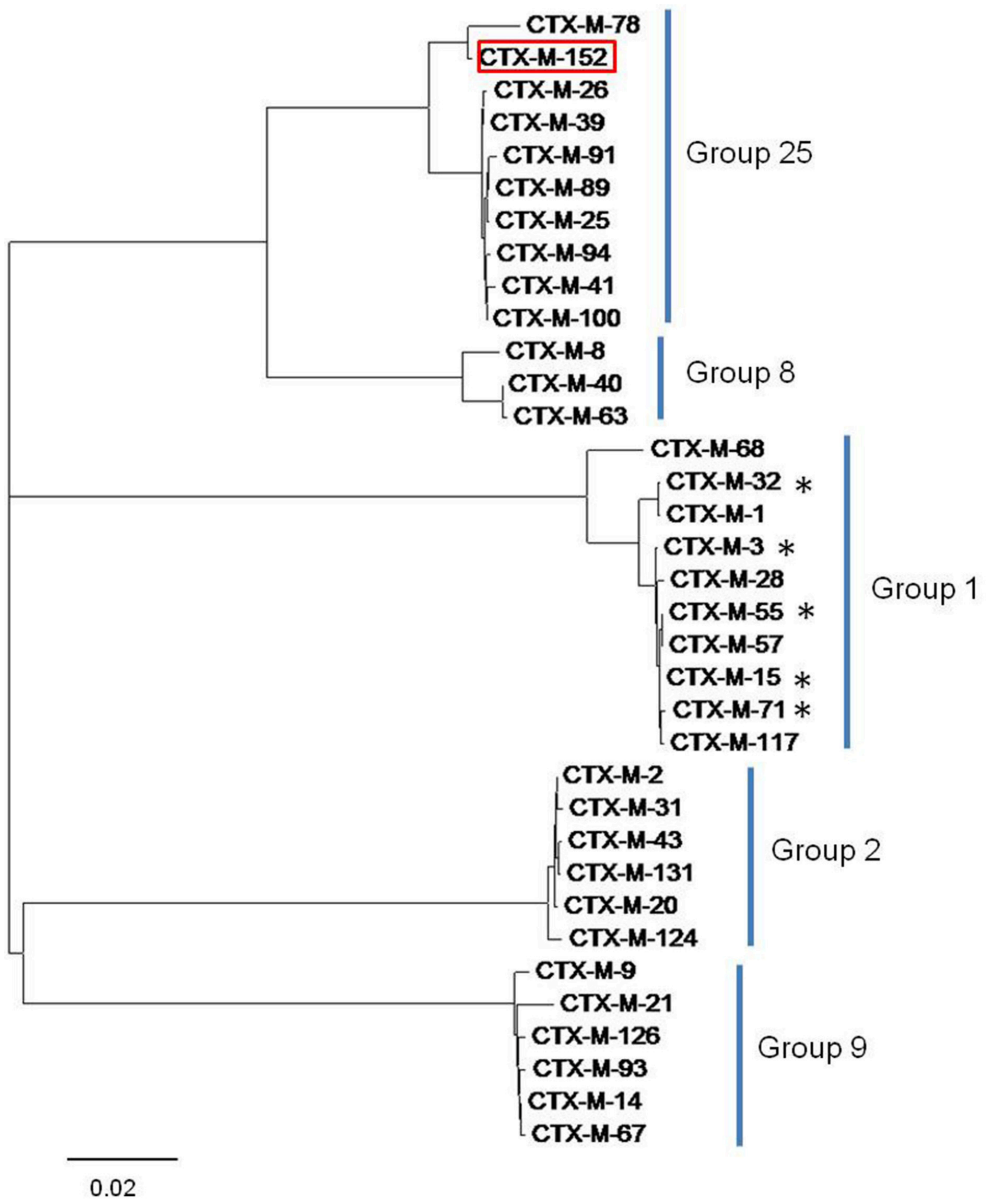
**Tree diagram showing similarity among the CTX-M lineage enzymes and clustering of different CTX-M group members**. The tree was constructed with the MEGA6 software. The variant CTX-M-152 is highlighted red and variants identified in this study are marked by asterisk.

Sequences of *bla*_TEM_ were found to be highly homologous to TEM-1 and TEM-116, while those corresponding to the *bla*_*CTX-M*_ gene displayed a disparity in their homology with five different members of the CTX-M-group-1 viz. CTX-M-3, CTX-M-15, CTX-M-32, CTX-M-55, and CTX-M-71 (Figure 2). The conserved motifs involved in cefotaxime hydrolysis found in CTX-M-1, were also conserved among variants obtained in the study (Figure S1). The nucleotide sequence of one variant of CTX-M gene denoted CTX-M-152 was found to be highly homologous with members of the CTX-M-group-25. Unlike CTX-M-1, CTX-M-25 like β-lactamases has been observed less frequently worldwide. Similar to our study, two other novel variants, CTX-M-94 and CTX-M-100 belonging to group-25 were reported in Israel (Vervoort et al., 2012). The *bla*_*CTX-M-152*_variant identified in *K. georgiana* isolate was found localized on chromosomal DNA while those identified in *E. coli* isolates screened from samples collected from downstream region of river Yamuna were found localized on plasmid. It strongly depicts their mobilization in the aquatic habitat. *K. georgiana* isolate harboring *bla*_*CTX-M-152*_ was found to have high MIC values against penicillins, cephalosporins, monobactams, and rifampicins (Table 3).

**Table 3:** **MIC values corresponding to different antibiotics for *K. georgiana* MRB7 isolate**.

**Antibiotic(s)**	**MIC (mg/L)**
Amikacin	0.5
Amoxycillin	>240
Aztreonam	120
Carbenicillin	>512
Cefotaxime	>512
Cefotaxime + Clavulanic Acid	0.38
Ceftazidime	256
Ceftazidime + Clavulanic Acid	1.5
Ceftriaxon	256
Ciprofloxacin	2
Kanamycin	3
Ofloxacin	0.1
Rifampicin	32
Tetracycline	12

Sequence of *bla*_*CTX-M-152*_ was found to be 98.6% similar with *bla*_*CTX-M-78*_ (CTX-M-group-25 member first identified in *K. georgiana*; Rodríguez et al., 2010). *bla*_*CTX-M-152*_ also showed 97.3% homology with the *bla*_*CTX-M-25*_ and *bla*_*CTX-M-94*_ sequences, 97.5% homology with *bla*_*CTX-M-39*_, and 97.4% homology with *bla*_*CTX-M-26*_, *bla*_*CTX-M-89*_, and *bla*_*CTX-M-100*_. Both variants (CTX-M-152 and CTX-M-78) differed from other members of the CTX-M-group-25 by amino acids substitutions M7I, C24Y, G81D, P99S, and G146D. CTX-M-152 is the only variant in the CTX-M-group-25 with a T154A substitution. This is the first report of any CTX-M variant containing histidine substituted with glutamine at position 26 (Figure S1). The genetic relatedness of the identified variant to members of group-25 obtained by MEGA6 suggests earlier branching of CTX-M-152, possibly due to Q26H, T154A, G89D, P99S, and D146G amino acid substitutions.

### SIFT analysis for deleterious substitutions

A homology check of the sequence revealed 10 individual substitutions (Q26H, Q89D, N92S, P99S, V103I, A120G, T189A, H197N, T209M, P266S) in sequence of CTX-M-152 in comparison to the most relevant CTX-M-9 with respect to sequence homology. To predict the functional importance of amino acid substitutions, all 10 substitutions were submitted independently to the SIFT programme to check their tolerance index with respect to CTX-M-9. Of the 10 substitutions, one having the T189A substitution was found to be deleterious, with a tolerance index score of 0.02, while others nine viz. Q29H, Q92D, N95S, P102S, V106I, A123G, H200N, T212M, and P269S are tolerable having tolerance index score of 0.12, 0.32, 0.64, 0.08, 0.17, 0.15, 0.50, 0.10, and 0.37, respectively.

### Homology modeling of CTX-M-152

**Figure 3:**
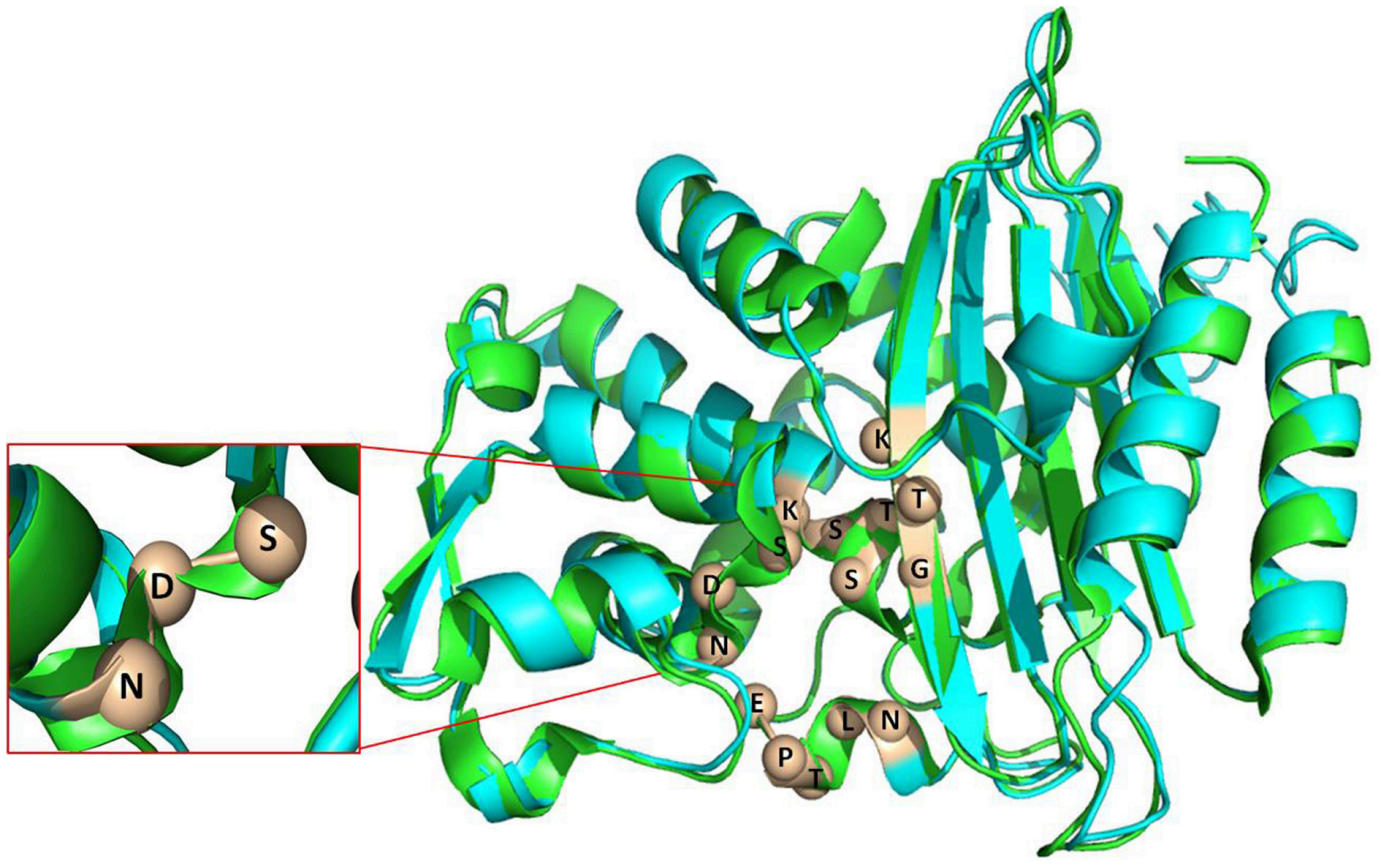
**Superimposed image of CTX-M-152(blue) and CTX-M-9 (green)**. The conserved element number 1 (S70-T71-S72-K73), conserved element number 2 (S130-D131-N132), conserved element number 3 (K234-T235-G236) and conserved element number 4 (E166-P167-T168-L169-N170) are labeled to show the active site.

When compared to the well-studied TEM, SHV and CTX-M-1 group members, there have been no structural studies of CTX-M-group-25 members to best of our knowledge. The nucleotide sequence of *bla*_*CTX-M-152*_ contains several point mutations (87G → C, 368C → G, 369A → G, 574A → G, and 576G → T), which lead to Q26H, A120G, and T189A amino acid substitutions, respectively. Upon finding close relatedness to CTX-M-9 (PDB 1YLJ), the backbone was used to generate the structural model of CTX-M-152. The structure generated by I-TASSER was monitored for stereochemical quality of the models using the PROCHECK and Rampage programs. As revealed by the Ramachandran contour plot obtained using the Rampage software, over 84% of the amino acid residues in the modeled structure were present in the most favored region, while another 10% were in the allowed region. The modeled structure contains an α and α/β domain, with the active site residing at the interface of two domains. Despite differences in orientation that include length of the helix and pleated regions, resemblance in the backbone structure of the two variants (CTX-M-9 and -152) shows an RMSD value (C^α^) of 0.412 Å (Figure 3). The pI value as calculated by Expasy tool was reported to be 8.81.

### Docking with cefotaxime

**Figure 4:**
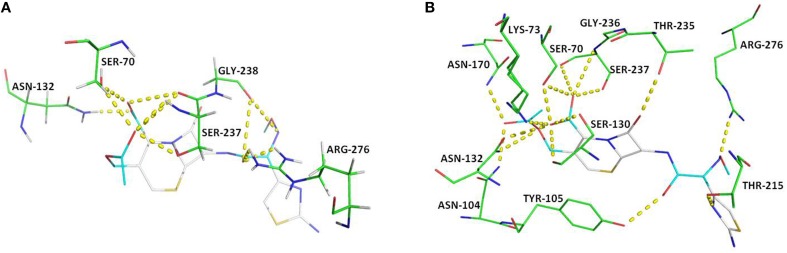
**Key polar interactions observed between enzyme CTX-M-152 (A) or CTX-M-9 (B) and Cefotaxime**. The broken lines represent hydrogen bonds and interacting amino acid residues are labeled. The cefotaxime is shown in stick form; oxygen atoms are red, nitrogen atoms in blue, carbon atoms white, and sulfur atoms in yellow.

**Figure 5:**
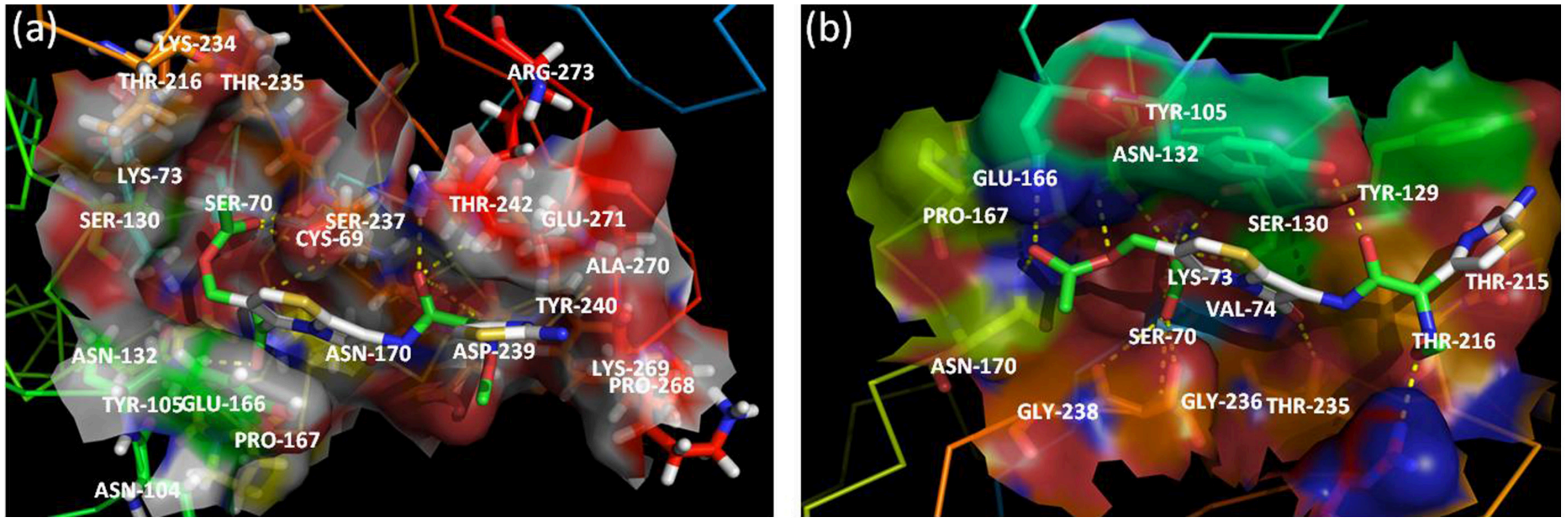
**Active site of CTX-M-152 (A) and CTX-M-9 (B) with substrate cefotaxime**. Residues around the binding pocket of substrate are depicted in transparent cloud form. The cefotaxime is presented in stick form; oxygen atoms are red, nitrogen atoms in blue, carbon atoms white, and sulfur atoms in yellow.

For docking purpose, a maximum of nine different conformations corresponding to cefotaxime (DB00497) were taken into account to estimate ligand binding conformations using the Lamarckian Genetic Algorithm (LGA) in the Auto dock. The conformation of the ligand with least binding energy indicates high affinity of β-lactamases for cefotaxime. Therefore, ligands showing a binding energy of -7.6 Kcal/mol that depict a more stable and effective interaction for facilitating the enzyme activity, was selected for further analysis. Although the docking results revealed interactions via seven H-bonds with five proposed active site residues (Ser70, Asn132, Ser237, Gly238, and Arg273), there were other surrounding residues that were also found to contribute toward hydrophobicity at the active site (Figures 4, 5). The active sites residues in association with the surrounding interacting residues are known to have four conserved regions that are critical to catalyze the substrate. The first conserved element (Ser70-Xxx-Xxx-Lys73) contains active serine70 and one helix turn with downstream Lys73, pointing to the bottom of the active site. Accordingly, we found that residues were similar, even in the structure of CTX-M-152. Drawz and Bonomo (2010) showed that CTX-M enzymes use this reactive serine (Ser70), a catalytic water molecule and an activator residue (Glu166) to hydrolyze the β-lactam ring through an acid-base catalytic mechanism (Drawz and Bonomo, 2010). Complying with the results of Chen et al. (2005a) regarding substitution of histidine for proline at position 99 in CTX-M-27, Pro99Ser substitution in CTX-M-152 was found to be in the tolerable range, thereby conferring no change in stability and function of CTX-M-152 (Chen et al., 2005a). The second motif, Ser130-Asp131-Asn132 situated on the short loop in the alpha domain forms the left side of the catalytic cavity. Among Ser130, Asp131, and Asn132 residues in the structure of CTX-M-152, only Asn132 interacted with cefotaxime. These findings are in accordance with studies suggesting residues in helixes and pleated regions (in the case of CTX-M-9) are favored over linker region residues for interaction with the substrate. Similarly, residue Asn104 and Tyr105 forms a bend in the binding site as observed in the structure of both CTX-M-9 and CTX-M-152. While working with the Toho-1-cefotaxime complex, Shimamura et al. (2002) reported involvement of the N104 side chain residue in hydrogen bonding with the side chain carbonyl of cefotaxime (Shimamura et al., 2002). However, changes in the hydrolytic pocket resulting from substitution of isoleucine for valine (V103I) render N104 residue out of range to form a hydrogen bond. The V103 residue conserved in CTX-M-1, -2, and -14 was replaced by isoleucine among members of CTX-M-group-25 in a similar method as reported for CTX-M-group-8. The conformational change influencing the positioning of Asn104 and/or Tyr105 greatly affects the interaction between enzymes and substrates. Based on the location of the third conserved element (Lys234-Thr235-Gly236) on the β3 strand of the β-sheet in the α/β domain, it forms the opposite wall of the catalytic cavity. Shakil and Khan (2010) reported the same type of interaction between side chains of cefotaxime and the backbone oxygen of Ser237 (Shakil and Khan, 2010). Upon docking of cefotaxime against the modeled CTX-M-152, only Ser237 of the Ser237 and Asn104 residues was found to interact with the carboxylate group of cefotaxime. This interaction might induce rotation of the carboxylate group in the acyl-intermediate structure of CTX-M, bringing the carbonyl group of β-lactam to a suitable position in the oxyanion hole, thereby promoting drug-hydrolysis. The last conserved element (Glu166-Pro167-Thr168-Leu169-Asn170) located on the 19-residue loop (positions 161–179), which is referred to as the omega (Ω) loop, forms the floor of active site. Both the β3 strand and the Ω-loop are important constituents of the active site cleft.

Among other residues that contribute to effective interaction between enzymes and antibiotics, Asn104, Ser237, Asp240, and Arg276, which form the flexible arms of the β3 strand and Ω-loop, were found to be involved in cefotaxime hydrolysing activity of CTX-M enzymes. As reported by Perez-Llarena et al. (2011), Ala219 in the loop that connects α and α/β domains (occupying top of active site), and are critical to the flexibility and breathing dynamics of β-lactamases, was also found conserved in CTX-M-152 (Perez-Llarena et al., 2011). In another study, Delmas et al. (2008) found that Val180, Arg191, Ala247, and Val260 constituted different hydrophobic clusters, thereby affecting the dynamics and flexibility of enzymes important to the hydrolysis of substrates (Delmas et al., 2008). Frequent among class A members, Arg274 residue increase substrate specificity because the side chain point toward the active site cavity. Similarly, residues Cys69, Ser72, Met135, Phe160 Thr165, and Ser237, which were conserved in CTX-M-152, are considered important with respect to substrate specificity (Péduzzi et al., 1997). In comparison to Asn270 residue of CTX-M-14 (along with other members of group-9) involved in establishing hydrogen bond with Asp240, CTX-M-152 similar to other members of group-25 possesses Lys270 to interact with residue Asp240 for correct positioning of β3 strand residues during catalytic process. CTX-M-152 containing Asp240 rather than Gly240 that increases its catalytic efficacy against ceftazidime, helped the variant to retain the high stability of the enzyme in the activity stability trade-off.

## Discussion

The escalating problem of multidrug resistance among infection causing organisms represents one of the greatest challenges worldwide. With increased antimicrobial usage, complexities in the resistance mechanisms have become more advanced. Densely populated centers with improper water supplies and inconsistent sanitation contribute significantly to acquisition and dissemination of resistance determinants among microbial inhabitants of water bodies. The Yamuna River, which originates from the Yamnotri glacier in the lower Himalayas (38°59′N 78°27′E), is the major source of water to urban areas in Delhi. Although the proportion of the river catchment area in Delhi is small (~2%), this area contributes more than 50% of pollutants that it receives through sewage from urban effluents, with high levels of antimicrobials in addition to toxic compounds being discharged by industries (Sharma and Kansal, 2011; Sehgal et al., 2012; Mutiyar and Mittal, 2014). The acquisition and transmission of resistance genes from microflora of human and animal origin discharged as part of sewage can substantially influence the pattern of resistance among the microbial inhabitants of the aquatic ecosystem (Amos et al., 2014).

Increasing incidences of ESBL-producing bacteria that showed a drastic shift in recent years in environmental settings are of serious concern. Contribution of selection to acquisition and as such spread of resistance among bacteria against major classes of antibiotics is alarming due to their higher dissemination rate. As such, high prevalence of ESBL producing isolates in natural water bodies like ponds, lakes, rivers, and tap water has drawn concern regarding increased spread of resistance in the environment (Upadhyay and Joshi, 2015). Recently, Bajaj et al. (2015) and Ahammad et al. (2014) also reported high prevalence of the several β–lactamase genes (TEM, SHV, CTX-M, AmpC, and NDM-1) among *E. coli* and other coliform bacterial species screened in collected water samples from upper ranges of Ganges River till its tributary Yamuna that stretches in Delhi and beyond. In our study, 93% of ESBL^+^ Gram negative isolates were observed to harbor *bla*_TEM_, *bla*_*CTX-M*_, and/or *bla*_OXY_. These variants were showing similarity to those reported by Wattal et al. (2010) and Rastogi et al. (2010), during their studies on ESBL production among clinical isolates (Rastogi et al., 2010; Wattal et al., 2010). In our study, we found ESBL producing bacterial isolates to have co-resistance to five others non-β-lactam classes of antibiotics in addition of exhibiting resistant phenotype to aztreonam (55%), ceftazidime (83%), cefazolin (85%), cefotaxime (73%), cefotetan (73%), and ertapenem (53%). The resistance of 21 isolates to ertapenem alerts for the decrease of carbapenem activity in the treatment of potential infections caused by these bacteria. These results are in concordance with the study of Datta et al. (2012), which reported steady increase in the percentage of carbapenem resistance among *E. coli* (40% in 2002 to 61% in 2009) and *K. pneumonia* (2% in 2002 to 52% in 2009) in a span of 10 years in tertiary-care hospital in New Delhi. In an another study, Center for Disease Dynamics, Economics & Policy (CDDEP) reported an increase in the percentage of carbapenems resistance from 10% in 2008 to 13% in 2013 among *E. coli* and 29% in 2008 to 57% in 2014 in *isolates of K. pneumonia* from India (Datta et al., 2012; Center for Disease Dynamics Economics and Policy (CDDEP), 2015). Besides strengthening the existing knowledge of their prevalence, presence of MDR, XDR, and PDR bacteria in the natural environment endorses them for the potent threat that they posses for the mankind. Mercury tolerance among the tested isolates appears to be an adaptation of bacteria that is correlated with their ability to live in mercury polluted environments. Accordingly, investigations of ESBL genes (*bla*_TEM_ and *bla*_*CTX-M*_) among bacteria that harbor both multidrug resistance and mercury tolerance are thought to provide useful information regarding their epidemiology in human influenced polluted environments.

Identification of *bla*_*CTX-M-152*_ in *K. georgiana* as part of this study is the first report of identification of any CTX-M-group-25 member from India. Docking studies of cefotaxime against modeled CTX-M-152 revealed that formation of a hydrogen bond between Ser237 and a carboxylate oxygen of cefotaxime induced rotation of the carboxylate group in the acyl-intermediate structure of CTX-M. Interaction of Ser237 with cefotaxime helps bring the carbonyl group of β-lactam to a suitable position in the oxyanion hole, thereby promoting hydrolysis of the drug. This was confirmed by the mutant S70G:S237A:R276A-cefotaxime complex, which displayed a significant loss of activity as a cefotaximase (Adamski et al., 2015). Simultaneously, Ser237 and Arg276, which are responsible for high substrate specificity, act cooperatively to promote cefotaxime hydrolysis through structural alterations in the active site to accommodate the larger cefotaxime molecule (Delmas et al., 2008, 2010). In a recent study, Adamski et al. (2015) revealed that co-operative interactions of Ser237 and Arg276 for cefotaxime enhance hydrolysis (~30-fold) relative to TEM-1/SHV-1 (Adamski et al., 2015). Hence, the presence of Ser237 and Arg276 in CTX-M-152 supports its high catalytic efficacy, even though it has a small active site similar to classical TEM-1 and SHV-1. Amino acid substitutions are of considerable significance in that they have direct or indirect involvement in changing enzyme activity. The hydrogen bond between side chains of Lys234 and Ser130 connecting α domain and α/β domain in class A enzymes was not observed in CTX-M-152. Conversely, substitutions such as N92S, P99S, A121G, and H197N, which represent changes with bulkier to light side chains, are thought to be involved in increasing the flexibility of protein, which is responsible for its high catalytic efficiency.

Delmas et al. (2008, 2010) reported binding of cefotaxime to CTX-M-9 that results in conformational changes at active sites though breakage of the hydrogen bond between Asn170 and Asp240 connecting the omega loop to the β3 strand (Delmas et al., 2008, 2010). This expands the active site to allow adequate positioning of the cefotaxime substrate for catalysis. The Asp240Gly substitution, which is known to increase the activity toward ceftazidime, was missing in CTX-M-152. Similar to Asp240Gly, the Val231Ala substitution on the β3 strand responsible for the stability-activity trade-off in the evolution of resistance enzymes, was absent from CTX-M-152. Although the two substitutions (Val231Ala and Asp240Gly) in the CTX-M enzymes did not alter the active site configuration, both have been reported to cause decreased protein stability, presumably through loss of favorable packing and polar interactions (Chen et al., 2005b). Hence, CTX-M-152 with residues having higher stability obtained from the Yamuna River isolate of *K. georgiana* is believed to be the progenitor of CTX-M genes. It is in concordance with the previous reports that documented the possible emergence of bla_*CTX-M*_ genes from *Kluyvera sp.* (Sarria et al., 2001; Humeniuk et al., 2002; Bonnet, 2004; Munday et al., 2004; McGettigan et al., 2009; Zhang et al., 2009).

Continuous threat posed by resistant organisms to human health has necessitated the need for further studies to improve understanding of their resistance mechanisms. Presence of CTX-M-152 with high stability and hydrolytic efficacy in an isolate of *K. georgiana* from the river Yamuna goes hand-on-hand with the generalization regarding natural environment acting as a source of resistance genes from which newer variants of enzymes evolve. Alongside, the presence of CTX-M-152 variant on the plasmid of *E. coli* isolates collected from downstream region of river Yamuna possibly demonstrate the mobilization of resistance genes through recombination events in bacteria. High incidence of CTX-M family member's warrant additional studies to be performed that might provide deeper insight into prevalence and information about the factors that led to spread of the resistant determinants, thereby can help in adopting strategies that can prevent selection, expansion, and transmission of resistance genes among bacteria associated with multiple human complications.

## Author contributions

QH conceived the topic. MA and AT contributed equally to this work. MA, AJ, and QH together contributed equally to the writing.

### Conflict of interest statement

The authors declare that the research was conducted in the absence of any commercial or financial relationships that could be construed as a potential conflict of interest.

## Supplementary material

Table S1 ***In vitro* susceptibility of ESBL^+^ bacterial isolates to 3^rd^ generation cephalosporins**.

Table S2 **Phenotypic disc confirmatory test of ESBL^+^ bacterial isolates**.

Table S3 **(A)** Susceptibilities of ESBL^+^ isolates to **β**-lactam class of antibiotics. **(B)** Susceptibilities of ESBL^+^ isolates to non-β-lactam classes of antibiotics.

Figure S1 **Multiple sequence alignment of CTX-M **β**-lactamases**. Amino acid sequences of representative members from five different groups of CTX-M ESBLs are aligned using CLUSTAL W program. Asterisks indicate identical amino acids. The four conserved domains of class A β-lactamases are marked with red line.
