# Supplementary material for: blaCTX-M-152, a Novel Variant of CTX-M-group-25, Identified in a Study Performed on the Prevalence of Multidrug Resistance among Natural Inhabitants of River Yamuna, India
Source: Front Microbiol. 2016 Feb 23;7:176. doi: 10.3389/fmicb.2016.00176 (PMC4762991; doi:10.3389/fmicb.2016.00176)
Supplement: Table S1 — In vitro susceptibility of ESBL+ bacterial isolates to 3rd generation cephalosporins. [file Table1.DOC]

| **Bacterial isolate** | **Zone of Inhibition (mm)** | | |
| --- | --- | --- | --- |
|  | **Ceftazidime (CAZ)**  **30µg/disc** | **Cefotaxime (CTX)**  **30µg/disc** | **Ceftriaxone (CTR)**  **30µg/disc** |
| *Klebsiella pneumoniae* MRA3 | 18 | 25 | 21 |
| *Aeromonas sps* MRA5 | 16 | 15 | 16 |
| *Aeromonas sps* MRA10 | 16 | 13 | 14 |
| *E.coli* MRA11 | 6 | 6 | 6 |
| *Klebsiella oxytoca* MRA13 | 10 | 18 | 20 |
| *E.coli* MRB2 | 21 | 25 | 25 |
| *E.coli* MRB6 | 10 | 7 | 6 |
| *Kluyvera georgiana* MRB7 | 15 | 24 | 22 |
| *E.coli* MRC2 | 15 | 7 | 5 |
| *E.coli* MRC3 | 17 | 8 | 9 |
| *E.coli* MRC6 | 12 | 10 | 10 |
| *E.coli* MRC7 | 20 | 24 | 25 |
| *E.coli* MRC13 | 17 | 14 | 10 |
| *E.coli* MRC17 | 6 | 9 | 8 |
| *E.coli* MRC24 | 20 | 14 | 13 |
| *E.coli* MRE2 | 16 | 10 | 8 |
| *E.coli* MRF6 | 23 | 26 | 28 |
| *Acinetobacter junii* MRH8 | 6 | 25 | 22 |
| *E.coli* MRK28 | 14 | 21 | 13 |
| *E.coli* MROB6 | 17 | 28 | 24 |
| *E.coli* MROB11 | 6 | 21 | 28 |
| *E.coli* MROB16 | 12 | 40 | 27 |
| *E.coli* MRAE2 | 6 | 9 | 6 |
| *E.coli* MRAE5 | 9 | 10 | 10 |
| *E.coli* MRAE6 | 6 | 6 | 6 |
| *E.coli* MRAE9 | 8 | 10 | 10 |
| *E.coli* MRAE14 | 18 | 23 | 23 |
| *E.coli* MRAE17 | 9 | 10 | 9 |
| *E.coli* MRAE18 | 6 | 6 | 6 |
| *E.coli* MRAE21 | 20 | 25 | 20 |
| *E.coli* MRAE23 | 10 | 10 | 9 |
| *E.coli* MRAE25 | 10 | 8 | 6 |
| *E.coli* MRAE26 | 10 | 6 | 6 |
| *E.coli* MRAE27 | 6 | 6 | 8 |
| *E.coli* MRAE31 | 6 | 6 | 0 |
| *E.coli* MRAE32 | 14 | 12 | 9 |
| *E.coli* MRAE33 | 9 | 8 | 0 |
| *E.coli* MRAE36 | 17 | 11 | 9 |
| *E.coli* MRAE42 | 6 | 0 | 0 |
| *E.coli* MRAE44 | 6 | 25 | 7 |
| *E.coli* ATCC 25922 | 21 | 29 | 26 |
| *Klebsiella pneumonia* ATCC 700603 | 9 | 16 | 27 |
