# Supplementary material for: blaCTX-M-152, a Novel Variant of CTX-M-group-25, Identified in a Study Performed on the Prevalence of Multidrug Resistance among Natural Inhabitants of River Yamuna, India
Source: Front Microbiol. 2016 Feb 23;7:176. doi: 10.3389/fmicb.2016.00176 (PMC4762991; doi:10.3389/fmicb.2016.00176)
Supplement: Table S2 — Phenotypic disc confirmatory test of ESBL+ bacterial isolates. [file Table2.DOC]

| **Bacterial Isolate** | **Zone of Inhibition (mm)** | | **+/-** | **Zone of Inhibition (mm)** | | **+/-** |
| --- | --- | --- | --- | --- | --- | --- |
|  | **CAZ** | **CAC** |  | **CTX** | **CEC** |  |
| *Klebsiella pneumoniae* MRA3 | 20 | 27 | + | 28 | 24 | - |
| *Aeromonas sps* MRA5 | 15 | 20 | + | 18 | 17 | - |
| *Aeromonas sps* MRA10 | 14 | 15 | - | 15 | 15 | - |
| *E.coli* MRA11 | 6 | 6 | - | 6 | 6 | - |
| *Klebsiella oxytoca* MRA13 | 9 | 22 | + | 22 | 27 | + |
| *E.coli* MRB2 | 20 | 26 | + | 28 | 29 | - |
| *E.coli* MRB6 | 8 | 18 | + | 6 | 18 | + |
| *Kluyvera georgiana* MRB7 | 26 | 30 | - | 31 | 30 | - |
| *E.coli* MRC2 | 14 | 21 | + | 13 | 20 | + |
| *E.coli* MRC3 | 13 | 21 | + | 9 | 20 | + |
| *E.coli* MRC6 | 9 | 20 | + | 9 | 19 | + |
| *E.coli* MRC7 | 21 | 27 | + | 27 | 26 | - |
| *E.coli* MRC13 | 16 | 24 | + | 12 | 22 | + |
| *E.coli* MRC17 | 6 | 6 | - | 6 | 6 | - |
| *E.coli* MRC24 | 15 | 21 | + | 15 | 21 | + |
| *E.coli* MRE2 | 12 | 21 | + | 7 | 19 | + |
| *E.coli* MRF6 | 6 | 6 | - | 15 | 18 | - |
| *Acinetobacter junii* MRH8 | 6 | 6 | - | 23 | 22 | - |
| *E.coli* MRK28 | 12 | 15 | - | 18 | 17 | - |
| *E.coli* MROB6 | 21 | 27 | + | 24 | 26 | - |
| *E.coli* MROB11 | 6 | 21 | + | 19 | 19 | - |
| *E.coli* MROB16 | 6 | 18 | + | 34 | 34 | - |
| *E.coli* MRAE2 | 6 | 13 | + | 9 | 12 | - |
| *E.coli* MRAE5 | 11 | 19 | + | 9 | 19 | + |
| *E.coli* MRAE6 | 8 | 18 | + | 7 | 17 | + |
| *E.coli* MRAE9 | 10 | 19 | + | 11 | 20 | + |
| *E.coli* MRAE14 | 18 | 21 | - | 23 | 21 | - |
| *E.coli* MRAE17 | 9 | 19 | + | 11 | 7 | - |
| *E.coli* MRAE18 | 6 | 6 | - | 6 | 6 | - |
| *E.coli* MRAE21 | 6 | 17 | + | 6 | 6 | - |
| *E.coli* MRAE23 | 11 | 18 | + | 10 | 8 | - |
| *E.coli* MRAE25 | 9 | 19 | + | 9 | 6 | - |
| *E.coli* MRAE26 | 6 | 16 | + | 6 | 6 | - |
| *E.coli* MRAE27 | 8 | 17 | + | 9 | 6 | - |
| *E.coli* MRAE31 | 6 | 6 | - | 6 | 6 | - |
| *E.coli* MRAE32 | 7 | 19 | + | 9 | 6 | - |
| *E.coli* MRAE33 | 6 | 18 | + | 9 | 6 | - |
| *E.coli* MRAE36 | 9 | 19 | + | 11 | 8 | - |
| *E.coli* MRAE42 | 8 | 18 | + | 8 | 17 | + |
| *E.coli* MRAE44 | 6 | 6 | - | 6 | 6 | - |
| *E.coli* ATCC 25922 | 21 | 25 | - | 22 | 22 | - |
| *Klebsiella pneumonia* ATCC 700603 | 9 | 20 | + | 16 | 16 | - |

**+** : test positive - : test negative

CAZ = **Ceftazidime** 30µg CAC = **Ceftazidime + Clavulanic Acid** 30µg + 10µg

CTX = **Cefotaxime** 30µg CEC = **Cefotaxime + Clavulanic acid** 30µg + 10µg
