# Supplementary material for: blaCTX-M-152, a Novel Variant of CTX-M-group-25, Identified in a Study Performed on the Prevalence of Multidrug Resistance among Natural Inhabitants of River Yamuna, India
Source: Front Microbiol. 2016 Feb 23;7:176. doi: 10.3389/fmicb.2016.00176 (PMC4762991; doi:10.3389/fmicb.2016.00176)
Supplement: Supplementary file 3 [file TableS3a.doc]

**Table S3a**: Susceptibilities of ESBL+ isolates to b-lactam class of antibiotics

| **Bacterial isolate** | **Penicillin** | | **Penicillin + inhibitor** | | **Monobactam** | | **Cephamycin** | | | | **Non-extended spectrum**  **Cephalosporins**  **I and 2^nd^ generation** | | | | **Carbapenems** | | | |
| --- | --- | --- | --- | --- | --- | --- | --- | --- | --- | --- | --- | --- | --- | --- | --- | --- | --- | --- |
|  | **AMP** | **S/I/R** | **A/S** | **S/I/R** | **AT** | **S/I/R** | **CTN** | **S/I/R** | **CX** | **S/I/R** | **CXM** | **S/I/R** | **CZ** | **S/I/R** | **ETP** | **S/I/R** | **IPM** | **S/I/R** |
| *Klebsiella pneumoniae* MRA3 | 6 | R | 16 | S | 25 | S | 21 | S | 22 | S | 20 | S | 23 | S | 24 | S | 25 | S |
| *Aeromonas sps* MRA5 | 6 | R | 14 | I | 23 | S | 6 | R | 18 | S | 14 | R | 18 | R | 28 | S | 25 | S |
| *Aeromonas sps* MRA10 | 6 | R | 9 | R | 20 | I | 6 | R | 19 | S | 7 | R | 8 | R | 28 | S | 20 | I |
| *E.coli* MRA11 | 6 | R | 9 | R | 8 | R | 6 | R | 6 | R | 6 | R | 6 | R | 17 | R | 20 | I |
| *Klebsiella oxytoca* MRA13 | 6 | R | 11 | R | 16 | R | 9 | R | 24 | S | 11 | R | 19 | R | 20 | I | 25 | S |
| *E.coli* MRB2 | 11 | R | 22 | S | 29 | S | 19 | S | 22 | S | 22 | S | 20 | I | 27 | S | 28 | S |
| *E.coli* MRB6 | 6 | R | 14 | I | 17 | R | 6 | R | 20 | S | 6 | R | 6 | R | 20 | I | 28 | S |
| *Kluyvera georgiana* MRB7 | 6 | R | 26 | S | 35 | S | 17 | S | 11 | R | 16 | I | 18 | R | 26 | S | 27 | S |
| *E.coli* MRC2 | 6 | R | 20 | S | 21 | S | 8 | R | 20 | S | 6 | R | 6 | R | 25 | S | 27 | S |
| *E.coli* MRC3 | 6 | R | 21 | S | 18 | I | 6 | R | 25 | S | 6 | R | 6 | R | 25 | S | 30 | S |
| *E.coli* MRC6 | 6 | R | 11 | R | 19 | I | 6 | R | 22 | S | 6 | R | 6 | R | 25 | S | 28 | S |
| *E.coli* MRC7 | 6 | R | 18 | S | 27 | S | 20 | S | 23 | S | 19 | S | 20 | I | 26 | S | 20 | I |
| *E.coli* MRC13 | 6 | R | 21 | S | 22 | S | 6 | R | 25 | S | 6 | R | 6 | R | 25 | S | 25 | S |
| *E.coli* MRC17 | 6 | R | 6 | R | 6 | R | 9 | R | 6 | R | 6 | R | 6 | R | 18 | R | 21 | I |
| *E.coli* MRC24 | 6 | R | 19 | S | 21 | S | 8 | R | 23 | S | 6 | R | 6 | R | 22 | S | 30 | S |
| *E.coli* MRE2 | 6 | R | 17 | S | 19 | I | 9 | R | 21 | S | 6 | R | 6 | R | 24 | S | 30 | S |
| *E.coli* MRF6 | 6 | R | 13 | I | 25 | S | 13 | I | 22 | S | 21 | S | 16 | R | 24 | S | 29 | S |
| *Acinetobacter junii* MRH8 | 28 | S | 25 | S | 6 | R | 6 | R | 12 | R | 6 | R | 17 | R | 13 | R | 30 | S |
| *E.coli* MRK28 | 13 | R | 19 | S | 25 | S | 24 | S | 18 | S | 12 | R | 10 | R | 27 | S | 33 | S |
| *E.coli* MROB6 | 6 | R | 18 | S | 10 | R | 22 | S | 17 | I | 17 | I | 17 | R | 17 | R | 30 | S |
| *E.coli* MROB11 | 6 | R | 25 | S | 9 | R | 23 | S | 16 | I | 17 | I | 16 | R | 16 | R | 29 | S |
| *E.coli* MROB16 | 6 | R | 16 | S | 10 | R | 23 | S | 17 | I | 18 | S | 20 | I | 18 | R | 27 | S |
| *E.coli* MRAE2 | 6 | R | 12 | I | 11 | R | 6 | R | 13 | R | 6 | R | 6 | R | 18 | R | 24 | S |
| *E.coli* MRAE5 | 6 | R | 14 | I | 16 | R | 6 | R | 18 | S | 6 | R | 6 | R | 19 | I | 24 | S |
| *E.coli* MRAE6 | 6 | R | 11 | R | 11 | R | 9 | R | 15 | I | 6 | R | 6 | R | 18 | R | 27 | S |
| *E.coli* MRAE9 | 6 | R | 17 | S | 15 | R | 6 | R | 15 | I | 6 | R | 6 | R | 17 | R | 25 | S |
| *E.coli* MRAE14 | 6 | R | 19 | S | 25 | S | 17 | S | 16 | I | 17 | I | 24 | S | 19 | I | 31 | S |
| *E.coli* MRAE17 | 6 | R | 14 | I | 17 | R | 6 | R | 18 | S | 6 | R | 11 | R | 18 | R | 29 | S |
| *E.coli* MRAE18 | 6 | R | 6 | R | 6 | R | 6 | R | 6 | R | 6 | R | 10 | R | 9 | R | 30 | S |
| *E.coli* MRAE21 | 6 | R | 6 | R | 28 | S | 19 | S | 16 | I | 6 | R | 20 | I | 19 | I | 33 | S |
| *E.coli* MRAE23 | 6 | R | 16 | S | 19 | I | 6 | R | 14 | R | 6 | R | 6 | R | 10 | R | 26 | S |
| *E.coli* MRAE25 | 6 | R | 6 | R | 17 | I | 6 | R | 10 | R | 6 | R | 6 | R | 9 | R | 33 | S |
| *E.coli* MRAE26 | 6 | R | 14 | I | 6 | R | 6 | R | 16 | I | 6 | R | 6 | R | 7 | R | 19 | R |
| *E.coli* MRAE27 | 6 | R | 8 | R | 15 | R | 6 | R | 15 | I | 6 | R | 6 | R | 10 | R | 13 | R |
| *E.coli* MRAE31 | 6 | R | 6 | R | 6 | R | 8 | R | 6 | R | 6 | R | 6 | R | 6 | R | 9 | R |
| *E.coli* MRAE32 | 6 | R | 6 | R | 14 | R | 11 | R | 15 | I | 6 | R | 6 | R | 11 | R | 20 | I |
| *E.coli* MRAE33 | 6 | R | 6 | R | 11 | R | 6 | R | 16 | I | 6 | R | 6 | R | 10 | R | 22 | I |
| *E.coli* MRAE36 | 6 | R | 6 | R | 15 | R | 6 | R | 16 | I | 6 | R | 6 | R | 10 | R | 23 | S |
| *E.coli* MRAE42 | 6 | R | 6 | R | 10 | R | 6 | R | 17 | I | 6 | R | 6 | R | 14 | R | 24 | S |
| *E.coli* MRAE44 | 6 | R | 6 | R | 8 | R | 6 | R | 6 | R | 6 | R | 6 | R | 12 | R | 24 | S |
| *E.coli* ATCC 25922 | 6 | R | 21 | S | 12 | R | 23 | S | 30 | S | 22 | S | 12 | R | 21 | I | 30 | S |
| *Klebsiella pneumonia* ATCC 700603 | 6 | R | 12 | I | 16 | R | 10 | R | 22 | S | 12 | R | 9 | R | 13 | R | 6 | R |

S : Sensitive I : Intermediate R : Resistant

AMP : Ampicillin (10µg) A/S : Ampicillin + Sulbactam (10/10µg) AT : Aztreonam (30µg) CTN : Cefotetan (30µg)

CX : Cefoxitin (30µg) CXM : Cefuroxime (30µg) CZ : Cefazolin (10µg) ETP : Ertapenem (10µg) IPM : Imipenem (10µg)
