# Supplementary material for: blaCTX-M-152, a Novel Variant of CTX-M-group-25, Identified in a Study Performed on the Prevalence of Multidrug Resistance among Natural Inhabitants of River Yamuna, India
Source: Front Microbiol. 2016 Feb 23;7:176. doi: 10.3389/fmicb.2016.00176 (PMC4762991; doi:10.3389/fmicb.2016.00176)
Supplement: Table S3 — (A) Susceptibilities of ESBL+ isolates to β-lactam class of antibiotics. (B) Susceptibilities of ESBL+ isolates to non-β-lactam classes of antibiotics. [file TableS3b.doc]

**Table S3b**: Susceptibilities of ESBL^+^ isolates to non-β-lactam classes of antibiotics.

| **Bacterial Isolate** | **Fluoroquinolones** | | | | | | **Polymixins** | | **Rifampicins** | | **Tetracyclins** | | **Amynoglycosides** | | | | **Trimethoprims** | |
| --- | --- | --- | --- | --- | --- | --- | --- | --- | --- | --- | --- | --- | --- | --- | --- | --- | --- | --- |
|  | **CIP** | **S/I/R** | **LE** | **S/I/R** | **OF** | **S/I/R** | **PB** | **S/I/R** | **RIF** | **S/I/R** | **TE** | **S/I/R** | **AK** | **S/I/R** | **TOB** | **S/I/R** | **TR** | **S/I/R** |
| *Klebsiella pneumoniae* MRA3 | 24 | I | 24 | S | 28 | S | 13 | S | 6 | R | 22 | S | 20 | S | 18 | S | 28 | S |
| *Aeromonas sps* MRA5 | 22 | I | 22 | S | 22 | S | 13 | S | 13 | R | 25 | S | 20 | S | 25 | S | 6 | R |
| *Aeromonas sps* MRA10 | 23 | I | 20 | S | 17 | S | 15 | S | 6 | R | 22 | S | 18 | S | 32 | S | 6 | R |
| *E.coli* MRA11 | 6 | R | 10 | R | 6 | R | 9 | R | 9 | R | 9 | R | 15 | I | 15 | S | 6 | R |
| *Klebsiella oxytoca* MRA13 | 20 | R | 20 | S | 11 | R | 14 | S | 10 | R | 23 | S | 19 | S | 25 | S | 6 | R |
| *E.coli* MRB2 | 28 | I | 25 | S | 20 | S | 14 | S | 9 | R | 22 | S | 18 | S | 28 | S | 28 | S |
| *E.coli* MRB6 | 17 | R | 20 | S | 16 | S | 13 | S | 6 | R | 22 | S | 15 | I | 18 | S | 27 | S |
| *Kluyvera georgiana* MRB7 | 25 | I | 25 | S | 21 | S | 9 | R | 6 | R | 12 | I | 16 | I | 18 | S | 6 | R |
| *E.coli* MRC2 | 23 | I | 21 | S | 19 | S | 13 | S | 7 | R | 20 | S | 18 | S | 22 | S | 27 | S |
| *E.coli* MRC3 | 21 | I | 22 | S | 20 | S | 14 | S | 6 | R | 21 | S | 17 | S | 22 | S | 28 | S |
| *E.coli* MRC6 | 6 | R | 12 | R | 6 | R | 15 | S | 7 | R | 8 | R | 18 | S | 15 | S | 6 | R |
| *E.coli* MRC7 | 24 | I | 14 | I | 21 | S | 14 | S | 10 | R | 25 | S | 15 | I | 20 | S | 6 | R |
| *E.coli* MRC13 | 10 | R | 14 | I | 15 | I | 7 | R | 8 | R | 25 | S | 18 | S | 20 | S | 6 | R |
| *E.coli* MRC17 | 8 | R | 10 | R | 6 | R | 11 | R | 6 | R | 20 | S | 18 | S | 19 | S | 23 | S |
| *E.coli* MRC24 | 26 | I | 24 | S | 21 | S | 12 | S | 10 | R | 23 | S | 18 | S | 18 | S | 26 | S |
| *E.coli* MRE2 | 8 | R | 12 | R | 6 | R | 12 | S | 7 | R | 20 | S | 18 | S | 17 | S | 23 | S |
| *E.coli* MRF6 | 25 | I | 22 | S | 18 | S | 12 | S | 8 | R | 11 | R | 18 | S | 13 | I | 6 | R |
| *Acinetobacter junii* MRH8 | 20 | R | 20 | S | 17 | S | 6 | R | 22 | S | 24 | S | 13 | R | 14 | I | 25 | S |
| *E.coli* MRK28 | 25 | I | 25 | S | 24 | S | 16 | S | 14 | R | 25 | S | 21 | S | 22 | S | 26 | S |
| *E.coli* MROB6 | 23 | I | 21 | S | 23 | S | 12 | S | 14 | R | 12 | I | 20 | S | 16 | S | 6 | R |
| *E.coli* MROB11 | 28 | I | 22 | S | 20 | S | 12 | S | 15 | R | 38 | S | 20 | S | 17 | S | 6 | R |
| *E.coli* MROB16 | 33 | S | 23 | S | 21 | S | 13 | S | 14 | R | 11 | R | 20 | S | 16 | S | 6 | R |
| *E.coli* MRAE2 | 17 | R | 23 | S | 17 | S | 17 | S | 10 | R | 24 | S | 16 | I | 14 | I | 22 | S |
| *E.coli* MRAE5 | 19 | R | 18 | S | 17 | S | 13 | S | 11 | R | 23 | S | 19 | S | 14 | I | 21 | S |
| *E.coli* MRAE6 | 18 | R | 22 | S | 17 | S | 18 | S | 11 | R | 12 | I | 22 | S | 16 | S | 24 | S |
| *E.coli* MRAE9 | 8 | R | 15 | I | 17 | S | 13 | S | 10 | R | 26 | S | 21 | S | 17 | S | 24 | S |
| *E.coli* MRAE14 | 25 | I | 24 | S | 17 | S | 12 | S | 13 | R | 26 | S | 22 | S | 16 | S | 28 | S |
| *E.coli* MRAE17 | 23 | I | 25 | S | 19 | S | 13 | S | 11 | R | 24 | S | 20 | S | 17 | S | 24 | S |
| *E.coli* MRAE18 | 6 | R | 11 | R | 6 | R | 15 | S | 10 | R | 10 | R | 17 | S | 14 | I | 6 | R |
| *E.coli* MRAE21 | 26 | I | 25 | S | 20 | S | 17 | S | 17 | I | 26 | S | 18 | S | 18 | S | 31 | S |
| *E.coli* MRAE23 | 23 | I | 25 | S | 24 | S | 15 | S | 11 | R | 25 | S | 18 | S | 16 | S | 25 | S |
| *E.coli* MRAE25 | 10 | R | 13 | R | 9 | R | 15 | S | 19 | I | 15 | S | 20 | S | 15 | S | 6 | R |
| *E.coli* MRAE26 | 17 | R | 22 | S | 17 | S | 11 | R | 10 | R | 22 | S | 20 | S | 14 | S | 10 | R |
| *E.coli* MRAE27 | 6 | R | 13 | R | 10 | R | 12 | S | 11 | R | 25 | S | 19 | S | 13 | I | 25 | S |
| *E.coli* MRAE31 | 6 | R | 11 | R | 6 | R | 15 | S | 9 | R | 8 | R | 6 | R | 6 | R | 27 | S |
| *E.coli* MRAE32 | 6 | R | 11 | R | 10 | R | 14 | S | 11 | R | 13 | I | 20 | S | 11 | R | 6 | R |
| *E.coli* MRAE33 | 18 | R | 17 | S | 15 | I | 12 | S | 7 | R | 19 | S | 18 | S | 15 | S | 6 | R |
| *E.coli* MRAE36 | 11 | R | 16 | I | 13 | I | 17 | S | 8 | R | 11 | R | 17 | S | 12 | R | 20 | S |
| *E.coli* MRAE42 | 15 | R | 18 | R | 17 | S | 12 | S | 6 | R | 22 | S | 20 | S | 12 | R | 6 | R |
| *E.coli* MRAE44 | 8 | R | 12 | R | 10 | R | 12 | S | 9 | R | 21 | S | 18 | S | 16 | S | 25 | S |
| *E.coli* ATCC 25922 | 35 | S | 24 | S | 31 | S | 13 | S | 10 | R | 22 | S | 10 | R | 21 | S | 24 | S |
| *Klebsiella pneumonia* ATCC 700603 | 23 | I | 19 | S | 19 | S | 13 | S | 6 | R | 13 | I | 6 | R | 16 | S | 18 | S |

S : Sensitive I : Intermediate R : Resistant

CIP : Ciprofloxacin (5µg) LE : Levofloxacin (5µg) OF : Ofloxacin (5µg) PB : Polymixin B (300 units) RIF : Rifampicin (5µg)

TE : Tetracyclin (30µg) AK : Amikacin (30µg) TOB : Tobramycin (10µg) TR : Trimethorim (5µg)
