# Supplementary material for: blaCTX-M-152, a Novel Variant of CTX-M-group-25, Identified in a Study Performed on the Prevalence of Multidrug Resistance among Natural Inhabitants of River Yamuna, India
Source: Front Microbiol. 2016 Feb 23;7:176. doi: 10.3389/fmicb.2016.00176 (PMC4762991; doi:10.3389/fmicb.2016.00176)
Supplement: Figure S1 — Multiple sequence alignment of CTX-M β-lactamases. Amino acid sequences of representative members from five different groups of CTX-M ESBLs are aligned using CLUSTAL W program. Asterisks indicate identical amino acids. The four conserved domains of class A β-lactamases are marked with red line. [file DataSheet1.doc]

10 20 30 40 50 60

....|....|....|....|....|....|....|....|....|....|....|....|

**CTX-M-1**  **MVKKSLRQFTLMATATVTLLLGSVPLYAQTADVQQKLAELERQSGGRLGVALINTADNSQ**

**CTX-M-15**  **----------------------------QTADVQQKLAELERQSGGRLGVALINTADNSQ**

**CTX-M-28**  **MVKKSLRQFTLMATATVTLLLGSVPLYAQTADVQQKLAELERQSGGRLGVALINTADNSQ**

**CTX-M-68**  **MVKKSLRQFTLMATATVTLLLGSVPLHAQTVDVQQKLAELERQSGGRLGVALINTADNSQ**

**CTX-M-117**  **MVKKSLRQFTLMATATVTLLLGSVPLYAQTADVQQKLAELERQSGGRLGVALINTADNSQ**

**CTX-M-31**  **MMTQSIRRSMLTVMATLPLLFSSATLHAQANSVQQQLEALEKSSGGRLGVALINTADNSQ**

**CTX-M-74**  **MMTQSIRRSMLTVMATLPLLFSSATLHAQANSVQQQLEALEKSSGGRLGVALINTADNSQ**

**CTX-M-124**  **MMTQSIRRSMLTVMATLPLLFSSATLHAQANSVQQQLEALEKSSGGRLGVALINTADNSQ**

**CTX-M-131**  **-MTQSIRRSMLTVMATLPLLFSSATLHAQANSVQQQLEALEKSSGGRLGVALINTADNSQ**

**CTX-M-40**  **-MRHRVKRMMLMTTACISLLLGSAPLYAQANDVQQKLAALEKSSGGRLGVALIDTADNAQ**

**CTX-M-63**  **-MRHRVKRMMLMTTACISLLLGSAPLYAQANDVQQKLAALEKSSGGRLGVALIDTADNAQ**

**CTX-M-8**  **MMRHRVKRMMLMTTACISLLLGSAPLYAQANDVQQKLAALEKSSGGRLGVALIDTADNAQ**

**CTX-M-14**  **----------------------------QTSAVQQKLAALEKSSGGRLGVALIDTADNTQ**

**CTX-M-21**  **MVTKRVQRMMFAGGAGIPLLLGSAPFYAQTSAGQQKLAALEKSSGGRLGVALIDTADNTQ**

**CTX-M-126**  **MVTKRVQRMMFAAAACIPLLLGSAPLYAQTSAVQQKLAALEKSSGGRLGVALIDTADNTQ**

**CTX-M-93**  **MVTKRVQRMMFAAAACIPLLLGSAPLYAQTSAVQQKLAALEKSSGGRLGVALIDTADNTQ**

**CTX-M-67**  **MVTKRVQRMMFAAAACIPLLLGSAPLYAQTSAVQQKLAALEKSSGGRLGVALIDTADNTQ**

**CTX-M-9**  **MVTKRVQRMMFAAAACIPLLLGSAPLYAQTSAVQQKLAALEKSSGGRLGVALIDTADNTQ**

**CTX-M-152**  **MMRKSVRRAILMTTACVSLLLASVPLYAHANDVQQKLAALEKSSGGRLGVALINTADNTQ**

**CTX-M-78**  **MMRKSVRRAILMTTACVSLLLASVPLYAQANDIQQKLAALEKSSGGRLGVALINTADNTQ**

**CTX-M-39**  **MMRKSVRRAMLMTTACVSLLLASVPLCAQANDVQQKLAALEKSSGGRLGVALINTADNTQ**

**CTX-M-100**  **MMRKSVRRAMLMTTACVSLLLASVPLCAQANDVQQKLAALEKSSGGRLGVALINTADNTQ**

**CTX-M-89**  **MMRKSVRRAMLMTTACVSLLLASVPLCAQANDVQQKLAALEKSSGGRLGVALINTADNTQ**

**CTX-M-26**  **MMRKSVRRAMLMTTACVSLLLASVPLCAQANDVQQKLAALEKSSGGRLGVALINTADNTQ**

**CTX-M-94**  **MMRKSVRRAMLMTTACVSLLLASVPLCAQANDVQQKLAALEKSSGGRLGVALINTADNTQ**

**CTX-M-25**  **MMRKSVRRAMLMTTACVSLLLASVPLCAQANDVQQKLAALEKSSGGRLGVALINTADNTQ**

**CTX-M-41**  **MMRKSVRRAMLMTTACVSLLLASVPLCAQANDVQQKLAALEKSSGGRLGVALINTADNTQ**

**CTX-M-91**  **MMRKSVRRAMLMTTACVSLLLASVPLCAQANDVQQKLAALEKSSGGRLGVALINTADNTQ**

**Clustal Consensus**  **:: **:* **:.**********:****:***

70 80 90 100 110 120

....|....|....|....|....|....|....|....|....|....|....|....|

**CTX-M-1**  **ILYRADERFAMCSTSKVMAVAAVLKKSESEPNLLNQRVEIKKSDLVNYNPIAEKHVDGTM**

**CTX-M-15**  **ILYRADERFAMCSTSKVMAAAAVLKKSESEPNLLNQRVEIKKSDLVNYNPIAEKHVNGTM**

**CTX-M-28**  **ILYRADERFAMCSTSKVMAAAAVLKKSESEPNLLNQRVEIKKSDLVNYNPIAEKHVNGTM**

**CTX-M-68**  **ILYRADERFAMCSTSKVMAAAAVLKKSESEPNLLNQRVEIKKSDLVNYNPIAEKHVNGTM**

**CTX-M-117**  **ILYRADERFAMCSTSKVMAAAAVLKKSESEPNLLNQRVEIKKSDLVNYNPIAEKHVNGTM**

**CTX-M-31**  **ILYRADERFAMCSTSKVMAAAAVLKQSESDKHLLNQRVEIKKSDLVNYNPIAEKHVNGTM**

**CTX-M-74**  **ILYRADERFAMCSTSKVMAAAAVLKQSESDKHLLNQRVEIKKSDLVNYNPIAEKHVNGTM**

**CTX-M-124**  **ILYRADERFAMCSTSKVMAAAAVLKQSESDKHLLNQRVEIKKSDLVNYNPIAEKHVNGTM**

**CTX-M-131**  **ILYRADERFAMCSTSKVMAAAAVLKQSESDKHLLNQRVEIKKSDLVNYNPIAEKHVNGTM**

**CTX-M-40**  **TLYRADERFAMCSTSKVMAAAAVLKQSETQKNVLSQKVEIKSSDLINYNPIAEKHVNGTM**

**CTX-M-63**  **TLYRADERFAMCSTSKVMAAAAVLKQSETQKNVLSQKVEIKSSDLINYNPIAEKHVNGTM**

**CTX-M-8**  **TLYRADERFAMCSTSKVMAAAAVLKQSETQKKVLSQKVEIKSSDLINYNPITEKHVNGTM**

**CTX-M-14**  **VLYRGDERFPMCSTSKVMAAAAVLKQSETQKQLLNQPVEIKPADLVNYNPIAEKHVNGTM**

**CTX-M-21**  **VLYRGDERFPMCSTSKVMAAAAVLKQSETQKQLLNQPVEIKPADLVNYNPIAEKHVNGTM**

**CTX-M-126**  **VLYRGDERFPMCSTSKVMAAAAVLKQSETQKQLLNQPVEIKPADLVNYNPIAEKHVNGTM**

**CTX-M-93**  **VLYRGDERFPMCSTSKVMAAAAVLKQSETQKQLLNQPVEIKPADLVNYNPIAEKHVNGTM**

**CTX-M-67**  **VLYRGDERFPMCSTSKVMAAAAVLKQSETQKQLLNQPVEIKPADLVNYSPIAEKHVNGTM**

**CTX-M-9**  **VLYRGDERFPMCSTSKVMAAAAVLKQSETQKQLLNQPVEIKPADLVNYNPIAEKHVNGTM**

**CTX-M-152**  **TLYRADERFAMCSTSKVMAAAAVLKQSETQKDLLSQRVEIKSSDLINYNPIAEKHVNGTM**

**CTX-M-78**  **TLYRADERFAMCSTSKVMAAAAVLKQSETQKDLLSQRVEIKSSDLINYNPIAEKHVNGTM**

**CTX-M-39**  **TLYRADERFAMCSTSKVMAAAAVLKQSETQKGLLSQRVEIKPSDLINYNPIAEKHVNGTM**

**CTX-M-100**  **TLYRADERFAMCSTSKVMAAAAVLKQSETQKGLLSQRVEIKPSDLINYNPIAEKHVNGTM**

**CTX-M-89**  **TLYRADERFAMCSTSKVMAVAAVLKQSETQKGLLSQRVEIKPSDLINYNPIAEKHVNGTM**

**CTX-M-26**  **TLYRADERFAMCSTSKVMAAAAVLKQSETQKGLLSQRVEIKPSDLINYNPIAEKHVNGTM**

**CTX-M-94**  **TLYRADERFAMCSTSKVMAAAAVLKQSETQKGLLSQRVEIKPSDLINYNPIAEKHVNGTM**

**CTX-M-25**  **TLYRADERFAMCSTSKVMAVAAVLKQSETQKGLLSQRVEIKPSDLINYNPIAEKHVNGTM**

**CTX-M-41**  **TLYRADERFAMCSTSKVMAAAAVLKQSETQKGLLSQRVEIKPSDLVNYNPIAEKHVNGTM**

**CTX-M-91**  **TLYRADERFAMCSTSKVMAVAAVLKQSETQKGLLSQRVEIKPSDLINYNPIAEKHVNGTM**

**Clustal Consensus**  *****.****.*********.*****:**:: :*.* **** :**:**.**:****:*****

130 140 150 160 170 180

....|....|....|....|....|....|....|....|....|....|....|....|

**CTX-M-1**  **SLAELSAAALQYSDNVAMNKLISHVGGPASVTAFARQLGDETFRLDRTEPTLNTAIPGDP**

**CTX-M-15**  **SLAELSAAALQYSDNVAMNKLIAHVGGPASVTAFARQLGDETFRLDRTEPTLNTAIPGDP**

**CTX-M-28**  **SLAELSAAALQYSDNVAMNKLIAHVGGPASVTAFARQLGDETFRLDRTEPTLNTAIPGDP**

**CTX-M-68**  **SLAELSAAALQYSDNVAMNKLIAHVGGPASVTAFARQLGDDTFRLDRTEPTLNTAIPGDP**

**CTX-M-117**  **SLAELSAAALQYSDNVAMNKLIAHVGGPASVTAFARQLGDETFRLDRTEPTLNTAIQGDP**

**CTX-M-31**  **TLAELGAAALQYSDNTAMNKLIAHLGGPDKVTAFARSLGDESFRLDRTEPTLNTAIPGDP**

**CTX-M-74**  **TLAELGAAALQYSDNTAMNKLIAHLGGPDKVTAFARSLGDETFRLDRTETTLNTAIPGDP**

**CTX-M-124**  **TLAELGAAALQYSDNTAMNKLIAHLGGPDKVTAFARSLGDETFRLDRTEPTLNTAIPGDP**

**CTX-M-131**  **TLAELGAAALQYSDNTAMNKLIAHLGGPDKVTAFARSLGDETFRLDRTEPTLNTAIPGDP**

**CTX-M-40**  **TLAELSAAALQYSDNTAMNKLIAHLGGPDKVTAFARAIGDDTFRLDRTEPTLNTAIPGDP**

**CTX-M-63**  **TLAELSAAALQYSDNTAMNKLIAHLGGPDKVTAFARAIGDDTFRLDRTEPTLNTAIPGDP**

**CTX-M-8**  **TLAELSAAALQYSDNTAMNKLIAHLGGPDKVTAFARAIGDNTFRLDRTEPTLNTAIPGDP**

**CTX-M-14**  **TLAELSAAALQYSDNTAMNKLIAQLGGPGGVTAFARAIGDETFRLDRTEPTLNTAIPGDP**

**CTX-M-21**  **TLAELSAAALQYSDNTAMNKLIAQLGGPGGVTAFARAIGDETFRLDRTEPTLNTAIPGDP**

**CTX-M-126**  **TLAELSAAALQYSDNTAMNKLIAQLGGPGGVTAFARAIGDETFRLDRTEATLNTAIPGDP**

**CTX-M-93**  **TLAELSAAALQYSDNTAMNKLIAQLGGPGGVTAFARAIGDETFRLDRTEPTQNTAIPGDP**

**CTX-M-67**  **TLAELSAAALQYSDNTAMNKLIAQLGGPGGVTAFARAIGDETFRLDRTEPTLNTAIPGDP**

**CTX-M-9**  **TLAELSAAALQYSDNTAMNKLIAQLGGPGGVTAFARAIGDETFRLDRTEPTLNTAIPGDP**

**CTX-M-152**  **TLGELSAAALQYSDNTAMNKLIAHLGGPGKVTAFARAIGDDTFRLDRTEPTLNTAIPGDP**

**CTX-M-78**  **TLGELSAAALQYSDNTAMNKLIAHLGGPGKVTAFARVIGDDTFRLDRTEPTLNTAIPGDP**

**CTX-M-39**  **TFGELSAAALQYSDNTAMNKLIAHLGGPDKVTAFARTIGDDTFRLDRTEPTLNTAIPGDP**

**CTX-M-100**  **TFGELSAAALQYSDNTAMNKLIAHLGGPDKVTAFARTIGDDTFRLDRTEPTLNTAIPGDP**

**CTX-M-89**  **TFGELSAAALQYSDNTAMNKLIAHLGGPDKVTAFARTIGDDTFRLDRTEPTLNTAIPGDP**

**CTX-M-26**  **TFGELSAAALQYSDNTAMNKLIAHLGGPDKVTAFARTIGDDTFRLDRTEPTLNTAIPGDP**

**CTX-M-94**  **TLGELSAAALQYSDNTAMNKLIAHLGGPDKVTAFARTIGDDTFRLDRTEPTLNTAIPGDP**

**CTX-M-25**  **TFGELSAAALQYSDNTAMNKLIAHLGGPDKVTAFARTIGDDTFRLDRTEPTLNTAIPGDP**

**CTX-M-41**  **TFGELIAAALQYSDNTAMNKLIAHLGGPDKVTAFARTIGDDTFRLDRTEPTLNTAIPGDP**

**CTX-M-91**  **TFGELSAAALQYSDNTAMNKLIAHLGGPDKVTAFARTIGDDTFRLDRTEPTLNTAIPGDP**

**Clustal Consensus** **::.** *********.******:::*** ****** :**::*******.* **** *****

190 200 210 220 230 240

....|....|....|....|....|....|....|....|....|....|....|....|

**CTX-M-1**  **RDTTSPRAMAQTLRNLTLGKALGDSQRAQLVTWMKGNTTGAASIQAGLPASWVVGDKTGS**

**CTX-M-15**  **RDTTSPRAMAQTLRNLTLGKALGDSQRAQLVTWMKGNTTGAASIQAGLPASWVVGDKTGS**

**CTX-M-28**  **RDTTSPRAMAQTLRNLTLGKALGDSQRAQLVTWMKGNTTGAASIQAGLPASWVVGDKTGS**

**CTX-M-68**  **RDTTSPRAMAQTLRNLTLGKALGDSQRAQLVTWMKGNTTGAASIQAGLPASWVVGDKTGS**

**CTX-M-117**  **RDTTSPRAMAQTLRNLTLGKALGDSQRAQLVTWMKGNTTGAASIQAGLPASWVVGDKTGS**

**CTX-M-31**  **RDTTTPLAMAQTLKNLTLGKALAETQRAQLVTWLKGNTTGSASIRAGLPKSWVVGDKTGS**

**CTX-M-74**  **RDTTTPLAMAQTLKNLTLGKALAETQRAQLVTWLKGNTTGSASIRAGLPKSWVVGDKTGS**

**CTX-M-124**  **RDTTTPLAMAQTLKNLTLGKALAETQRAQLVTWLKGNTTGSASIRAGLPKSWVVGDKTGS**

**CTX-M-131**  **RDTTTPLAMAQTLKNLTLGKALAETQRAQLVTWLKGNTTGSASIRAGLPKSWVVGDKTGS**

**CTX-M-40**  **RDTTTPLAMAQTLRHLTLGSALGETQRAQLVTWLKGNTTGAASIQAGLPTSWVVGDKTGS**

**CTX-M-63**  **RDTTTPLAMAQTLRHLTLGSALGETQRAQLVTWLKGNTTGAASIQAGLPTSWVVGDKTGS**

**CTX-M-8**  **RDTTTPLAMAQTLRNLTLGSALGETQRAQLVTWLKGNTTGAASIQAGLPTSWVVGDKTGS**

**CTX-M-14**  **RDTTTPRAMAQTLRQLTLGHALGETQRAQLVTWLKGNTTGAASIRAGLPTSWTVGDKTGS**

**CTX-M-21**  **RDTTTPRAMAQTLRQLTLGHALGETQRAQLVTWLKGNTTGAASIRAGLPTSWTVGDKTGS**

**CTX-M-126**  **RDTTTPRAMAQTLRQLTLGHALGETQRAQLVTWLKGNTTGAASIRAGLPTSWTVGDKTGS**

**CTX-M-93**  **RDTTTPRAMAQTLRQLTLGHALGETQRAQLVTWLKGNTTGAASIRAGLPTSWTVGDKTGS**

**CTX-M-67**  **RDTTTPRAMAQTLRQLTLGHALGETQRAQLVTWLKGNTTGAASIRAGLPTSWTVGDKTGS**

**CTX-M-9**  **RDTTTPRAMAQTLRQLTLGHALGETQRAQLVTWLKGNTTGAASIRAGLPTSWTAGDKTGS**

**CTX-M-152**  **RDTTTPLAMAQALRNLTLGNALGDTQRAQLVMWLKGNTTGAASIQAGLPTSWVVGDKTGS**

**CTX-M-78**  **RDTTTPLAMAQTLRNLTLGNALGDTQRAQLVTWLKGNTTGAASIQAGLPTSWVVGDKTGS**

**CTX-M-39**  **RDTTTPLAMAQALRNLTLGNALGDTQRAQLVMWLKGNTTGAASIQAGLPTSWVVGDKTGS**

**CTX-M-100**  **RDTTTPLAMAQALRNLTLGNALGDTQRAQLVMWLKGNTTGAASIQAGLPTSWVVGDKTGS**

**CTX-M-89**  **RDTTTPLAMAQALRNLTLGNALGDTQRAQLVMWLKGNTTGAASIQAGLPTSWVVGDKTGS**

**CTX-M-26**  **RDTTTPLAMAQALRNLTLGNALGDTQRAQLVMWLKGNTTGAASIRAGLPTSWVVGDKTGS**

**CTX-M-94**  **RDTTTPLAMAQALRNLTLGNALGDTQRAQLVMWLKGNTTGAASIQAGLPTSWVVGDKTGS**

**CTX-M-25**  **RDTTTPLAMAQALRNLTLGNALGDTQRAQLVMWLKGNTTGAASIQAGLPTSWVVGDKTGS**

**CTX-M-41**  **RDTTTPLAMAQALRNLTLGNALGDTQRAQLVMWLKGNTTGAASIQAGLPTSWVVGDKTGS**

**CTX-M-91**  **RDTTTPLAMAQSLRNLTLGNALGDTQRAQLVMWLKGNTTGAASIQAGLPTSWVVGDKTGS**

**Clustal Consensus** ******:* ****:*::**** **.::****** *:******:***:**** **..********

250 260 270 280 290

....|....|....|....|....|....|....|....|....|....|.

**CTX-M-1**  **GDYGTTNDIAVIWPKDRAPLILVTYFTQPQPKAESRRDVLASAAKIVTNGL**

**CTX-M-15**  **GGYGTTNDIAVIWPKDRAPLILVTYFTQPQPKAESRRDVLASAAKIVTDGL**

**CTX-M-28**  **GGYGTTNDIAVIWPKDRAPLILVTYFTQPQPKAESRRDVLASAAKIVTNGL**

**CTX-M-68**  **GDYGTTNDIAVIWPKDRAPLILVTYFTQPQPKAESRRDVLASAAKIVTDGL**

**CTX-M-117**  **GGYGTTNDIAVIWPKDRAPLILVTYFTQPQPKAESRRDVLASAAKIVTDGL**

**CTX-M-31**  **GDYGTTNDIAVIWPENHAPLVLVTYFTQPEQKAESRRDILAAAAKIVTHGF**

**CTX-M-74**  **GDYGTTNDIAVIWPENHAPLVLVTYFTQPEQKAESRRDILAAAAKIVTHGF**

**CTX-M-124**  **GDYGTTNDIAIIWPENHAPLVLVTYFTQPEQKAESRRDVLAAAAKIVTHGF**

**CTX-M-131**  **GGYGTTNDIAVIWPENHAPLVLVTYFTQPEQKAESRRDILAAAAKIVTHGF**

**CTX-M-40**  **GDYGTTNDIAVIWPEGRAPLILVTYFTQPEQKAESRRDVLAAAAKIVTDGY**

**CTX-M-63**  **GDYGTTNDIAVIWPEGRAPLILVTYFTQPEQKAENRRDVLAAAAKIVTDGY**

**CTX-M-8**  **GDYGTTNDIAVIWPEGRAPLILVTYFTQPEQKAESRRDVLAAAAKIVTDGY**

**CTX-M-14**  **GDYGTTNDIAVIWPQGRAPLVLVTYFTQPQQNAESRRDVLASAARIIAEGL**

**CTX-M-21**  **GDYGTTNDIAVIWPQGRAPLVLVTYFTQPQQNAESRRDVLASAARIIAEGL**

**CTX-M-126**  **GDYGTTNDIAVIWPQGRAPLVLVTYFTQPQQNAESRRDVLASAARIIAEGL**

**CTX-M-93**  **GGYGTTNDIAVIWPQGRAPLVLVTYFTQPQQNAESRRDVLASAARIIAEGL**

**CTX-M-67**  **GDYGTTNDIAVIWPQGRAPLVLVTYFTQPQQNAESRRDVLASAARIIAEGL**

**CTX-M-9**  **GDYGTTNDIAVIWPQGRAPLVLVTYFTQPQQNAESRRDVLASAARIIAEGL**

**CTX-M-152**  **GDYGTTNDIAVIWPEGRAPLVLVTYFTQSEPKAESRRDVLAAAARIVTDGY**

**CTX-M-78**  **GDYGTTNDIAVIWPEGRAPLVLVTYFTQPEPKAESRRDVLAAAARIVTDGY**

**CTX-M-39**  **GDYGTTNDIAVIWPEGRAPLVLVTYFTQSEPKAESRRDVLAAAARIVTDGY**

**CTX-M-100**  **GGYGTTNDIAVIWPEGRAPLVLVTYFTQSEPKAESRRDVLAAAARIVTDGY**

**CTX-M-89**  **GDYGTTNDIAVIWPEGRAPLVLVTYFTQSEPKAESRRDVLAAAARIVTDGY**

**CTX-M-26**  **GDYGTTNDIAVIWPEGRAPLVLVTYFTQSEPKAESRRDVLAAAARIVTDGY**

**CTX-M-94**  **GGYGTTNDIAVIWPEGRAPLVLVTYFTQSEPKAESRRDVLAAAARIVTDGY**

**CTX-M-25**  **GGYGTTNDIAVIWPEGRAPLVLVTYFTQSEPKAESRRDVLAAAARIVTDGY**

**CTX-M-41**  **GGYGTTNDIAVIWPEGRAPLVLVTYFTQSEPKAESRRDVLAAAARIVTDGY**

**CTX-M-91**  **GDYGTTNDIAVIWPEGRAPLVLVTYFTQSEPKAESRRDVLAAAARIVTDGY**

**Clustal Consensus** ***.********:***:.:***:*******.: :**.***:**:**:*::.***

Figure S1: Multiple sequence alignment of CTX-M β-lactamases. Amino acid sequences of representative members from five different groups of CTX-M ESBLs are aligned using CLUSTAL W program. Asterisks indicate identical amino acids. The four conserved domains of class A β-lactamases are marked with red line.
